# Metabarcoding Reveals Lacustrine Picocyanobacteria Respond to Environmental Change Through Adaptive Community Structuring

**DOI:** 10.3389/fmicb.2021.757929

**Published:** 2021-11-12

**Authors:** Lena A. Schallenberg, John K. Pearman, Carolyn W. Burns, Susanna A. Wood

**Affiliations:** ^1^Department of Zoology, University of Otago, Dunedin, New Zealand; ^2^Coastal and Freshwater Group, Cawthron Institute, Nelson, New Zealand

**Keywords:** picocyanobacteria, *Cyanobium*, *Synechococcus*, 16S rRNA, community structure, metabarcoding

## Abstract

Picocyanobacteria (Pcy) are important yet understudied components of lake foodwebs. While phylogenetic studies of isolated strains reveal a high diversity of freshwater genotypes, little is known about abiotic drivers associated with Pcy in different lakes. Due to methodological limitations, most previous studies assess potential drivers using total cell abundances as a response, with often conflicting and inconsistent results. In the present study, we explored how picocyanobacterial communities respond to environmental change using a combination of epifluorescence microscopy and community data determined using 16S rRNA gene metabarcoding. Temporal shifts in picocyanobacterial abundance, diversity and community dynamics were assessed in relation to potential environmental drivers in five contrasting lakes over 1year. Cell abundances alone were not consistently related to environmental variables across lakes. However, the addition of metabarcoding data revealed diverse picocyanobacterial communities that differed significantly between lakes, driven by environmental variables related to trophic state. Within each lake, communities were temporally dynamic and certain amplicon sequence variants (ASVs) were strongly associated with specific environmental drivers. Rapid shifts in community structure and composition were often related to environmental changes, indicating that lacustrine Pcy can persist at high abundances through collective community adaptation. These results demonstrate that a combination of microscopy and metabarcoding enables an in-depth characterisation of picocyanobacterial communities and reveals strain-specific drivers. We recommend that future studies cease referring to picocyanobacterial as one functional group and take strain specific variability into consideration.

## Introduction

Picocyanobacteria (Pcy) are increasingly being recognised as important components of freshwater ecosystems (Stockner, 1991; Caroppo, 2015). Forming the base of lacustrine microbial foodwebs, Pcy contribute significantly to primary productivity and their relative contribution is thought to increase with decreasing trophic state (Petersen, 1991; Stockner and Shortreed, 1991; Callieri and Stockner, 2002). Most work to date has focused on populations in oligo-and mesotrophic lakes, however recent studies suggest that the importance of Pcy in eutrophic and hypertrophic lakes may have been overlooked (Carrick and Schelske, 1997; Jasser et al., 2010; Wood et al., 2017; Schallenberg et al., 2021). Pcy are now known to inhabit a wide range of freshwater and brackish environments, likely due to their high adaptability and phenotypic plasticity (Callieri, 2017; Huber et al., 2017).

While the diversity and dynamics of marine Pcy (largely marine *Synechococcus* and *Prochlorococcus*) have been well-studied, freshwater Pcy have received less attention (Callieri and Stockner, 2002). New technologies are revealing the presence and abundance of diverse Pcy strains in a range of freshwater environments (Ruber et al., 2016; Wood et al., 2017). However, little is known about the temporal diversity of Pcy across lakes of varying type and trophic state, and whether the environmental drivers that control Pcy dynamics differ among lakes.

Unravelling the complex abiotic drivers of species composition and density is difficult. To date, most studies exploring environmental drivers have used data on cell abundances and pigment type and not considered changes in community composition. These studies often produce inconsistent and conflicting results, adding to the uncertainty around which factors are important in controlling freshwater Pcy abundances and shaping communities. A review of studies which have assessed environmental drivers of Pcy community dynamics illustrates the difficulty in determining not only broad scale but also local population drivers (Table 1). For example, within oligotrophic lakes, total phosphorus (TP) is both positively (Stockner and Shortreed, 1991) and negatively (Schallenberg and Burns, 2001; Zhong et al., 2013) correlated with Pcy abundances (Table 1). Similarly, within eutrophic and hypertrophic lakes, temperature has been shown to be both positively (Szelag-Wasielewska, 2004; Pulina et al., 2017) and negatively (Huber et al., 2017) correlated with Pcy abundance (Table 1). Therefore, it is not surprising that large-scale spatial studies, including those that assess lakes across a trophic gradient, often find insignificant correlations (e.g., Lavallée and Pick, 2002; Table 1).

**Table 1:** Studies correlating picocyanobacterial abundances or communities with environmental drivers and the resulting significant correlations reported.

Lake	Trophic state	Abundance vs. env. variable correlations	Community vs. env. variable correlations	Analysis technique	References
Lakes Wanaka and Wakatipu, New Zealand	Oligotrophic/Experimental	Inconsistent results: N and P addition.		Epifluorescence microscopy	Bayer, 2013
Baltic Sea (transect)	Oligotrophic/Marine		Different OTU’s correlated differently with variables.	16S rRNA 454-pyrosequencing	Bertos-Fortis et al., 2016
Six New Zealand lakes	Range	Positive: temp		Epifluorescence microscopy	Burns and Stockner, 1991
Forty-Five New Zealand lakes	Range	Positive: Secchi depth negative: ciliate biomass, DOC, DRP, TSS		Epifluorescence microscopy	Burns and Galbraith, 2007
Lake Chaohu, China	Eutrophic	Read abundance=positive: DIN:DIP and NH_4_-N		16S rRNA clone libraries	Cai and Kong, 2013
Lake Maggiore, Italy	Oligotrophic		Temp, mixing depth and pH correlated with change in composition	Epifluorescence microscopy and ARISA	Callieri et al., 2012a
Lake Balaton, Hungary	Meso-eutrophic		Different OTU’s correlated differently with variables.	16S rRNA and cpcBA-IGS clone libraries	Felföldi et al., 2011
Lake Chascomus, Argentina	Hypertrophic	Negative: temp	None	Epifluorescence microscopy and 16S and 16S-23S ITS clone libraries	Huber et al., 2017
Forty-Eight Quebec lakes	Range	None.		Epifluorescence microscopy	Lavallée and Pick, 2002
Fifty-Seven Canadian lakes	Range	Positive: P (up to 15 ug/L) Negative: P (>15 ug/L)		Epifluorescence microscopy	Pick, 2000
Three Mediterranean lagoons	Eutrophic	Positive: temp Positive and negative: N and P		Epifluorescence microscopy	Pulina et al., 2017
Ostersee Lakes, Bavaria	Range		OTU’s not correlated with trophic state or environmental variables.	16S rRNA Illumina sequencing	Ruber et al., 2016, 2018
Lake Wakatipu, New Zealand	Oligotrophic/Experimental	Negative: TP addition		Epifluorescence microscopy	Schallenberg and Burns, 2001
Kennedy Lake, Canada	Oligotrophic/Experimental	Positive: TP+TN addition		Epifluorescence microscopy	Stockner and Shortreed, 1988
Eleven Canadian lakes	Oligotrophic	Positive: TP		Epifluorescence microscopy	Stockner and Shortreed, 1991
Lake Strzeszynskie, Poland	Eutrophic	Colonies positively correlated with temp		Epifluorescence microscopy	Szelag-Wasielewska, 2004
Lake Tahoe, USA	Oligotrophic	Positive: nitrocline, depth		Epifluorescence microscopy	Winder, 2009
Seven reservoirs, Singapore	-	Negative: nutrients, turbidity, salinity. Positive: DO		Flow cytometry	Yang et al., 2019
Lake Annecy and Bourget, France	Oligo- and mesotrophic	Bourget=positive: Temp Negative: N and P annecy=none		Flow cytometry, 16S rRNA DGGE	Zhong et al., 2013

N, nitrogen; TN, total nitrogen; P, phosphorus; TP, total phosphorus; Temp, temperature; DOC, dissolved organic carbon; DRP, dissolved reactive phosphorus; TSS, total dissolved solids; DIP:DIP, dissolved inorganic nitrogen:dissolved inorganic phosphorus; NH_4_-N, ammoniacal nitrogen; and OTUs, operational taxonomic units.

This variation in identified drivers could be due to differences in the types and locations of lakes, although research on larger planktonic cyanobacteria, especially bloom forming cyanobacteria, suggests drivers are similar at a global scale (Harke et al., 2016; Huisman et al., 2018). A more likely scenario is that due to their small size and associated issues with identification beyond family level, Pcy have historically been studied and reported as one group (“Picocyanobacteria” or “autotrophic picoplankton”), failing to acknowledge the diversity and varied functional abilities of Pcy (Cabello-Yeves et al., 2018; Sánchez-Baracaldo et al., 2019; Di Cesare et al., 2020). While total cell abundances are important for understanding the contribution of Pcy to primary productivity, shifts or differences in community composition might better reflect changes in biotic and abiotic drivers.

Diverse communities of Pcy have now been found in freshwater and brackish lakes using genetic fingerprinting and barcoding techniques (Sanchez-Baracaldo et al., 2008; Callieri et al., 2012a; Ruber et al., 2016; Pulina et al., 2017; Wood et al., 2017; Schallenberg et al., 2021), suggesting that freshwater Pcy diversity is as high or higher than that of their marine counterparts (Sanchez-Baracaldo et al., 2008). Studies employing these techniques have found that different Pcy operational taxonomic units (OTU’s) can respond differently to the same driver, indicating the potential for community level responses to drivers. For example, Felföldi et al. (2011) found different Pcy OTU’s responded differently to drivers such as temperature (Table 1). Further, Bertos-Fortis et al. (2016) found that the most abundant *Synechococcus* OTU’s identified in Baltic Sea samples correlated in often-opposite ways with the same environmental variable. Studies from the marine environment also highlight distinct niche specificity of Pcy strains across horizontal and vertical transects (Stockner and Shortreed, 1988; West and Scanlan, 1999; Johnson et al., 2006; Six et al., 2007). Callieri et al. (2012a) showed that this was also the case in Lake Maggiore, where distinct vertical partitioning of Pcy OTU’s was found during stratification. With an often-greater diversity of strains inhabiting lacustrine compared to marine environments, it is possible that spatial and temporal community shifts may exhibit a stronger response to environmental change than overall cell abundance.

In the present study we used epifluorescence microscopy and community composition data from DNA metabarcoding (targeting the 16S ribosomal RNA gene; 16S rDNA) to assess temporal Pcy abundances, diversity and dynamics in five contrasting lakes over a 12-month period. This dataset allowed us to explore drivers of spatial and temporal changes in Pcy across a diversity of lake types while investigating the complementarity and effectiveness of these two methodologies. We hypothesised that: (i) Cell abundances within lakes will be temporally dynamic but not consistently related to environmental drivers across the lakes or trophic states, suggesting that strain-level responses may be occurring; (ii) Lacustrine Pcy will be diverse, with different communities inhabiting different lakes due to drivers related to trophic state; (iii) Pcy communities will differ temporally within lakes driven by shifts in physico-chemical variables; and (iv) Pcy communities in deep, stratified lakes will show vertical niche partitioning due to differing epi- and hypolimnetic conditions, suggesting vertical niche partitioning. Overall, we theorised that the combined use of microscopy and metabarcoding techniques will provide an enhanced picture of Pcy dynamics and responses to physico-chemical change.

## Materials and Methods

### Study Lakes

**Figure 1:**
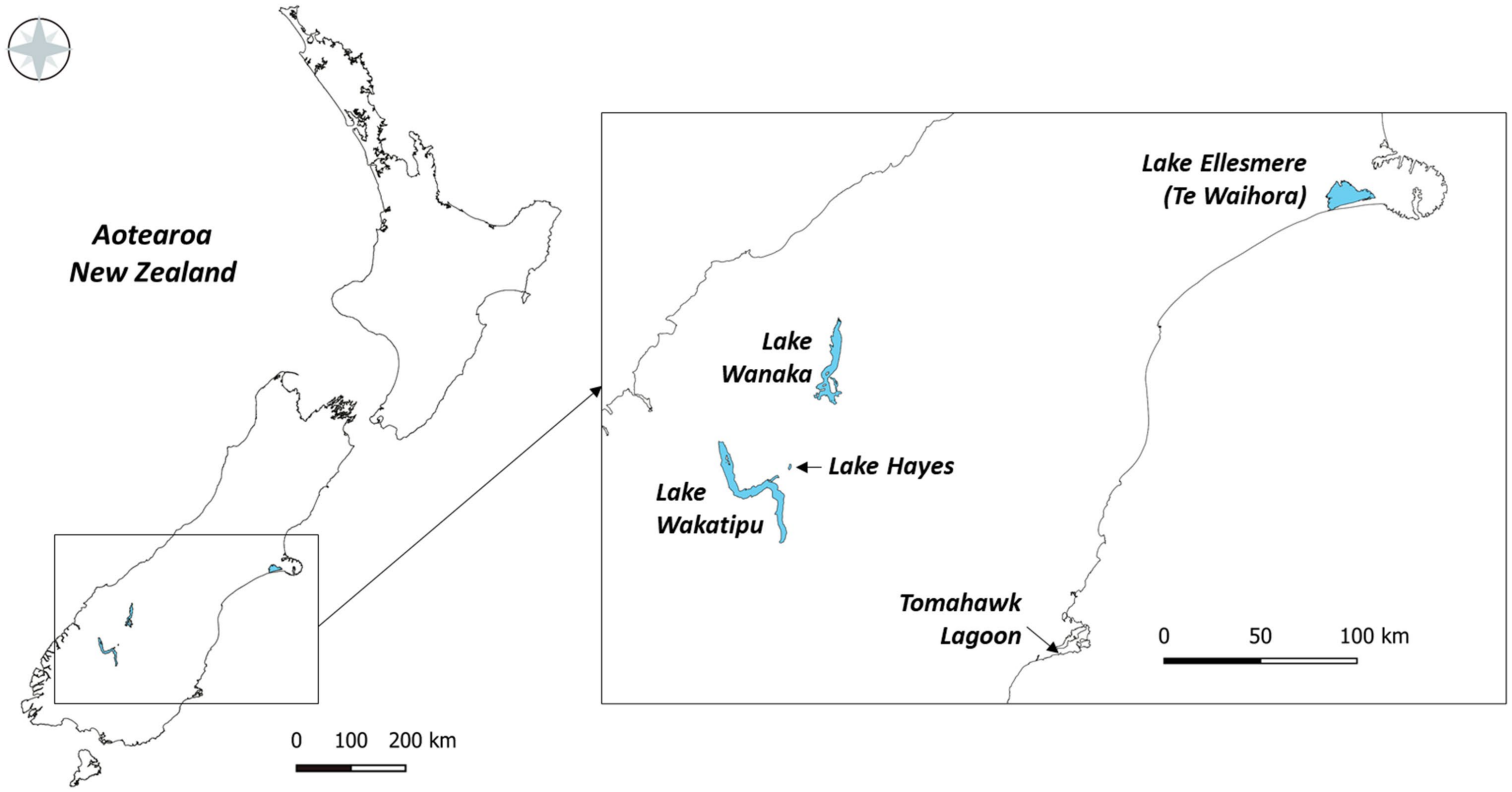
The five lakes sampled from February 2018 to February 2019 in the South Island of New Zealand.

Five lakes in southern New Zealand known to contain Pcy (Wood et al., 2017) were sampled monthly for 1year (February 2018 to February 2019; Figure 1). These lakes represent different lake types, trophic states, morphologies, and salinities (Table 2). Lakes Wanaka and Wakatipu are large, deep, oligotrophic, typically monomictic peri-alpine lakes (Table 2). Lake Hayes is a small, typically monomictic, eutrophic lake that experiences both clear and turbid water phases. Tomahawk Lagoon is a small, eutrophic, urban coastal lagoon without direct connection to the sea. Lake Ellesmere/Te Waihora is a large, supertrophic, Intermittently Closed and Open Lagoon Lake (ICOLL) which is opened to the sea multiple times per year (Table 2).

**Table 2:** Lake characteristics and typical water quality parameters.

	Wanaka	Wakatipu	Hayes	Tomahawk	Ellesmere
Trophic state	Microtrophic (TLI=1.7)	Oligotrophic (TLI=2.1)	Eutrophic (TLI=4.7)	Eutrophic	Supertrophic (TLI=6.8)
Lat/long	44.647°S 169.100°E	45.065°S 168.685°E	44.979°S 168.809°E	45.901°S 170.549°E	43.789°S 172.466°E
Lake type	Glacial	Glacial	Glacial	Coastal	ICOLL
Lake area (km^2^)	180	289	2.7	0.1	197.6
Maximum depth (m)	311	380	33	1.2	3
TN (mg/m^3^)	53	53	345	-	2,200
TP (mg/m^3^)	1	1	39	-	210
Mixing	Monomictic	Monomictic	Monomictic		Polymictic

TN and TP are 5-year medians (2016–2019). All data retrieved from the Land and Water Aotearoa database (www.lawa.co.nz). TLI, trophic level index; ICOLL, Intermittently Closed and Open Lagoon Lake; TN, total nitrogen; and TP, total phosphorus.

### Sample Collection

All lakes were sampled monthly in the main basin. In the monomictic lakes (Wanaka, Wakatipu, and Hayes), both epilimnion and hypolimnion samples were taken. Composite samples were taken from the epilimnion in Lakes Wanaka and Wakatipu that were comprised of equal volumes of water from 0.5, 10, 15, 30, and 45m depths, and hypolimnion samples were taken from 150m deep. In Lake Hayes, epilimnion samples were combined samples from 0.5, 2.5, 5, and 7.5m depths while the hypolimnion samples were from 22m. Samples from Lakes Wanaka, Wakatipu and Hayes were collected using a clean Van Dorn sampler before being transferred into sterile 1L Nalgene™ bottles. In shallow Lake Ellesmere/Te Waihora and Tomahawk Lagoon, samples were taken from 0.5m depth using a sterile, submerged 1L Nalgene™ bottle. All samples were placed in the dark immediately and transported on ice to the laboratory within 24h and subsampled for nutrient analysis, epifluorescence microscopy and DNA extraction.

### Water Quality

Temperature (°C), dissolved oxygen (DO %) and salinity (ppt) depth profiles were recorded from Lakes Wanaka, Wakatipu, and Hayes using a SeaCAT CTD (SBE 19plus V2, Sea-Bird Electronics, WA, United States). The average value taken across the combined epilimnion depth range was used, to correspond with the depth of the epilimnion water sample taken. In Lake Ellesmere/Te Waihora and Tomahawk Lagoon, temperature, DO and salinity were measured using a water quality sonde (YSI, OH, United States), at 0.5m depth. Secchi depth was measured at all lakes except for Tomahawk Lagoon.

Nutrients measured were total nitrogen (TN), TP, dissolved reactive phosphorus (DRP), nitrate-nitrite nitrogen (NNN) and ammoniacal nitrogen (NH_4_-N). Samples (50ml) for measurement of dissolved nutrients (NNN, NH_4_-N, and DRP) were filtered (GF/F, Whatman, United Kingdom) using an acid washed syringe. Nutrient samples from Lakes Ellesmere/Te Waihora, Wanaka, Wakatipu, and Hayes were analysed using a Continuous Flow Analyser at Hill Laboratories following APHA 4500 methods (Hamilton, New Zealand) with the following limits of detection; 1μgL^−1^ (DRP and NNN), 4μgL^−1^ (TP), 5μgL^−1^ (NH_4_-N), and 10μgL^−1^ (TN). Chlorophyll-*a* (Chl-*a*) analysis for these lakes was undertaken using subsamples of up to 500ml of water following APHA 10200 H methods at the same laboratory with a detection limit of 0.00002g/m^3^. Nutrients in Tomahawk lagoon samples were measured on a Continuous Flow Analyser (San++, Skalar Analytical, Breda, The Netherlands) using standard colorimetric methods with the following limits of detection; 0.1μgL^−1^ (TN) and 0.5μgL^−1^ (TP, DRP, NH_4_-N, and NNN). Samples for Chl-*a* analysis in Tomahawk Lagoon were filtered (<200ml; GF/F, Whatman, United Kingdom) and the filters immediately frozen (−20°C) until later extraction using 90% alkaline aqueous acetone. Chl-*a* was measured spectrophotometrically following Wetzel and Likens (1991) using a UV mini 1,240 Spectrophotometer (Shimadzu, Japan).

### Epifluorescence Microscopy

Cell abundances were enumerated using epifluorescence microscopy following the protocol described in Schallenberg et al. (2021). Briefly, 15ml subsamples of water from Lakes Wanaka, Wakatipu, Hayes and Tomahawk and 0.1ml from Lake Ellesmere/Te Waihora (diluted with 9.9ml Milli-Q water) were each filtered onto black polycarbonate filters (0.2μm Cyclopore, Whatman, United States) using a 1.2μm backing filter to ensure even distribution. Filters were viewed using a Zeiss Axiophot microscope (Carl Zeiss Jena GmbH, Jena, Germany) equipped with a narrow-banded green filter set CY3 (Ex 520–570, DM 565, BA 535–675). Using this filter set, phycoerythrin-rich picocyanobacteria autofluoresce bright yellow while phycocyanin-rich picocyanobacteria autofluoresce bright red (Jasser and Callieri, 2017). Individual cells, colonies, and the number of cells per colony were counted from at least 20 fields of view or 400 individual cells at 1000× magnification.

### DNA Extraction and 16S Ribosomal RNA Gene Sequencing

Water samples were filtered in triplicate (150ml for Lakes Wanaka, Wakatipu, and Hayes; 30ml for Lake Ellesmere/Te Waihora; 50ml for Tomahawk Lagoon) using sterile filters (0.22μm, S-Pak, Sigma, United States) and immediately frozen (−20°C). DNA extraction, PCR amplification and sequencing of Pcy sequences was carried out following the methodology described in Schallenberg et al. (2021). Briefly, DNA was extracted from filters using a PowerSoil® DNA Isolation Kit (QIAGEN, United States) including one negative extraction control every 23rd sample. PCR was performed using cyanobacteria-specific primers CYB359-F 5'-GGGGAATYTTCCGCAATGGG-3' and CYB784-R 5'-ACTACWGGGGTATCTAATCCC-3' (Nübel et al., 1997) which target the V3–V4 region [~380 base pairs (bp)] of the cyanobacterial 16S rRNA gene. PCR conditions are outlined in Schallenberg et al. (2021). PCR products were purified using an Agencourt® AMPure® XP Kit (Beckman Coulter, CA, United States) before being diluted to 5ngμl^−1^ and submitted to Auckland Genomics Limited (Auckland, New Zealand) for library preparation and sequencing. Library preparation followed the Illumina 16S metagenomics library prep manual. A second round of PCR added dual indexes to the samples and 5μl of each subsequent PCR reaction was pooled before a single clean-up was undertaken. The pooled library was quality controlled using a bioanalyzer and the library was diluted to 4nM, denatured, and diluted to a final concentration of 7 ρM with a 15% PhiX spike. An Illumina MiSeq was used to produce paired end sequences (2×250bp) which have been deposited in the NCBI short read database under BioProject accession number: PRJNA749894.

### Bioinformatics

Forward and reverse reads were assigned to samples after automatic demultiplexing using MiSeq Reporter (v2). Primers were eliminated from sequences allowing for one mismatch per sequence using Cutadapt (Martin, 2011). The remaining bioinformatic analysis was conducted in R version 4.0.4 (R Core Team, 2021). The DADA2 package version 1.16.0 (Callahan et al., 2016) was used to filter and trim forward and reverse reads to 230 and 228bp with 2 and 4 maximum errors, respectively. Error profiles for the forward and reverse reads were assessed using a parametric model utilising 1×10^8^ bases. Sequences were then dereplicated and a pseudo-pooling approach was used to infer amplicon sequence variants (ASVs). Sequences were merged, allowing one mismatch, with a minimum overlap of 10bp followed by chimera removal using the default consensus approach. Taxonomy was assigned using the SILVA 138 database (Quast et al., 2013) by employing an RDP Naïve Bayesian Classifier with a bootstrap threshold of 50 (Wang et al., 2007). The *phyloseq* package version 1.32.0 (McMurdie and Holmes, 2013) was used for data pre-processing and statistical analysis.

Chloroplasts and non-cyanobacterial sequences were removed, and the resulting cyanobacterial sequences filtered to include only ASVs with more than five reads that were present in more than one sample. This approach was chosen as low abundance ASVs were not the focus of this study and given the inclusion of triplicate samples, if an ASV was present in only one triplicate, it is likely to be erroneous. The distribution of sample sequencing depth at various processing steps is provided in Supplementary Figure S1 and a table of sequencing depths for each sample is provided in Supplementary Table S1. Triplicate samples were then merged after confirming they clustered together on a non-metric multidimensional scaling (NDMS) ordination.

### Statistical Analysis

All statistical analysis was carried out using R version 4.0.4 (R Core Team, 2021) and Microsoft Excel (Excel version 2016). Bioinformatic and statistical analysis scripts can be found at https://github.com/lenaschall/Temporal_Picocyanobacteria_Scripts. Spearman rank correlations were calculated between total cell abundance and individual environmental variables for each sample. To test for significant differences in colonial cell contributions between the epi- and hypolimnion of the monomictic lakes, *t*-tests were conducted. Metabarcoding samples were normalised by randomly subsampling without replacement, to an even sequencing depth (4,000 reads) following assessment of a rarefaction curve plotted using the ggrare function from the ranacapa package (Kandlikar et al., 2018), confirming most samples had reached a plateau at this level (Supplementary Figure S2). Due to the low number of cyanobacterial reads in some samples, 24 samples and an associated 12 ASVs were discarded from the statistical analysis. For the remaining analysis of Pcy communities, rarefied data were used and only ASVs from the Order Synechococcales were retained.

Observed richness along with Shannon and Simpson alpha diversity metrics were calculated for each lake using the *phyloseq* package and differences in Shannon diversity between lakes was assessed using ANOVA. A permutational multivariate analysis of variance (PerMANOVA) was used to test for significant differences in picocyanobacterial communities between lakes using the adonis function from the *vegan* package (Oksanen et al., 2020). The assumption of homogeneous dispersions between groups was tested between lakes using the betadisper function from the *vegan* package. To assess temporal community structure between months within each lake, reads were converted to relative abundances and rare ASVs (contributing on average <1% of sample reads) were combined into one category. Pcy community structure was analysed using stacked bar plots in the R package *ggplot2* version 3.3.5 (Wickham, 2016).

Canonical analyses of principal coordinates (CAP) were performed on the square-root transformed ASV data using both the presence/absence of ASVs (binary Jaccard) and total read abundance (weighted Jaccard) to assess differences in Pcy communities between the five lakes, and within each lake. For analysis between the five lakes, environmental variables related to all lakes were included together in the analysis, while the remaining analyses were conducted separately for each lake. Environmental variables were first analysed for collinearity and pairs returning a correlation coefficient >0.7 were scrutinised (Dormann et al., 2013), with the least biologically relevant variables removed from further analysis (Supplementary Table S2). The bioenv function from the package *vegan* was used to find the optimal subset of environmental variables that maximise the rank correlation with community dissimilarities. The Variance Inflation Factors (VIF) of the Bioenv selected variables were calculated to confirm variable independence before inclusion in the CAP model (Supplementary Table S2). The variables were then included as CAP constraints to determine the influence of the optimised set of environmental variables on Pcy community structure across months, using the weighted Jaccard dissimilarity matrices. Individual ASVs were plotted on the CAP, with abundant ASVs labelled, to assess relationships between ASVs and environmental constraints. To assess significance of the models, a PerMANOVA was calculated on the CAP ordinations using the Adonis function in *vegan* to determine if the constraints, environmental variables and CAP axes explained a significant amount of the variation in community dissimilarities.

To test for significant differences in the vertical structuring of Pcy communities in Lakes Wanaka and Wakatipu, a PerMANOVA was designed with depth as a factor. To determine if nutrient concentrations differed significantly with depth, nutrients measured from corresponding epi- and hypolimnetic samples from each monomictic lake were analysed using two-tailed *t*-tests.

## Results

### Temporal Abundance and Environmental Correlations

**Figure 2:**
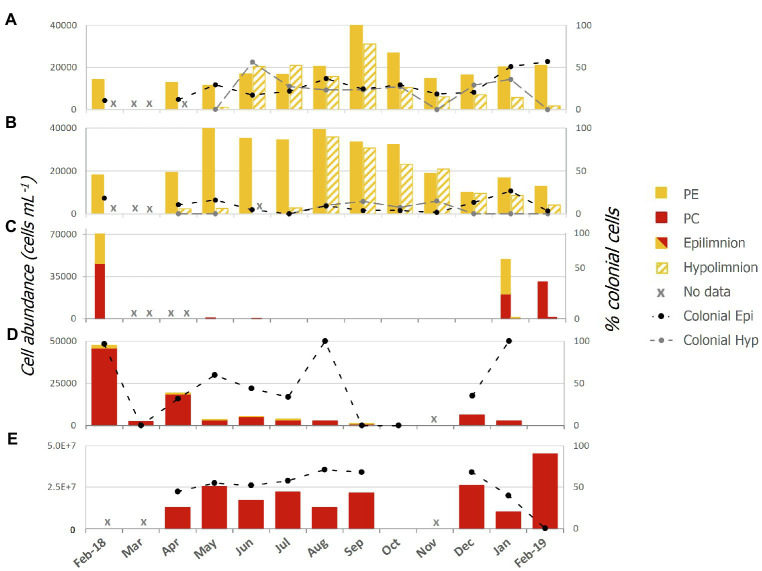
Picocyanobacterial abundances from February 2018 to February 2019 in lakes; **(A)** Wanaka, **(B)** Wakatipu, **(C)** Hayes, **(D)** Tomahawk Lagoon, and **(E)** Ellesmere/Te Waihora as determined using epifluorescence microscopy. Colonial cells (as a percentage of the total cell count) are indicated by the black and grey thatched lines and secondary axis. Red bars represent phycocyanin (PC) rich cells while yellow bars represent phycoerythrin (PE) rich cells. Epi, epilimnion and Hyp, hypolimnion.

Abundances of single celled and colonial Pcy were variable over the sampling period in all lakes (Figure 2; Supplementary Table 3). In oligotrophic Lake Wanaka, peak abundances were recorded in spring (September, October, and November) in both the epi- and hypolimnion (Figure 2A). In oligotrophic Lake Wakatipu, bimodal autumn (March, April, and May) and winter (June, July, and August) peaks occurred in the epilimnion with peak hypolimnetic abundances occurring in Winter (August; Figure 2B). Winter destratification is reflected by a large increase in cell abundances in the hypolimnion (150m depth) of Lakes Wanaka and Wakatipu in June and August, respectively (Supplementary Figure S3). Colonial cells in Lake Wanaka accounted for an average of 25% of total cells over the year, while colonial cells in Lake Wakatipu accounted for 7% on average. A *t*-test revealed no significant difference between the percent contribution of colonial cells in the epi- and hypolimnion of Lakes Wanaka (*F*=0.54, *p*=0.47) and Wakatipu (*F*=2.15, *p*=0.15).

In the two eutrophic lakes (Lake Hayes and Tomahawk Lagoon), both PE and PC-rich Pcy were found, with PE cells more common in Lake Hayes than Tomahawk Lagoon (Figures 2C,D). Pcy were not consistently present in these two lakes, with cell numbers in Lake Hayes peaking in Summer 2018 (January and February) but absent from July 2018 to January 2019 (Figure 2C). No colonial cells were found in Lake Hayes. Pcy in Tomahawk lagoon reached a peak abundance in February 2018 (47,543 cells ml^−1^; Figure 2D). Tomahawk Lagoon had the highest variation in colonial cells, accounting for between 0 and 100% of total cells throughout the year. Supertrophic Lake Ellesmere/Te Waihora had a consistently high number of Pcy cells throughout the year, with a peak in February 2019 of 4.5×10^7^ cells ml^−1^ (Figure 2E). The cells in Lake Ellesmere/Te Waihora consisted of single-cell and colonial PC-rich morphotypes, with colonial cells occurring in groups of four. Colonial cells contributed an average of 51% to the total cell count over the year, however a sharp decline in colonial cells occurred in February 2019 correlated with the largest peak in overall abundance (Figure 2E).

Spearman’s rank correlations between cell abundances (both total cells and the percentage of colonial cells) and corresponding water quality parameters revealed a number of significant correlations, both positive and negative (Table 3). No consistent correlations were found across all lakes or within oligotrophic or eutrophic lakes. Total hypolimnetic cell abundances in Lakes Wanaka and Wakatipu and colonial cells in Lake Wakatipu were significantly negatively correlated with NNN while in Lake Ellesmere/Te Waihora NNN was positively correlated with colonial cells (Table 3). In the Lake Wakatipu epilimnion, total cell abundances were negatively correlated with TP but positively correlated with DRP. The contribution of colonial cells was significantly positively correlated with TN and DO in Tomahawk Lagoon and Lake Ellesmere/Te Waihora, respectively.

**Table 3:** Spearman’s rank correlations between total and colonial (as a percentage of total) cell abundances and environmental variables for each lake.

Lake		Total cells			% Colonial cells		
		Variable	*ρ*	*p*	Variable	*ρ*	*p*
Wanaka	Epilimnion						
	*Hypolimnion*	NNN	−0.85	0.016^*^			
Wakatipu	*Epilimnion*	TP	−0.85	0.034^*^			
		DRP	0.88	0.021^*^			
	*Hypolimnion*	NNN	−0.89	0.007^**^	NNN	−0.84	0.017^*^
Hayes	*Epilimnion*						
	*Hypolimnion*						
Ellesmere					NNN	0.68	0.045^*^
					DO	0.67	0.050^*^
Tomahawk					TN	0.93	0.008^**^

TN, total nitrogen; NNN, nitrate-nitrite nitrogen; NH_4_-N, ammoniacal nitrogen; DO, dissolved oxygen; and DRP, dissolved reactive phosphorus.

* Significant at *α*=0.05.

** Significant at *α*=0.01.

### Diversity and Dynamics of Picocyanobacterial Communities Between Lakes

**Figure 3:**
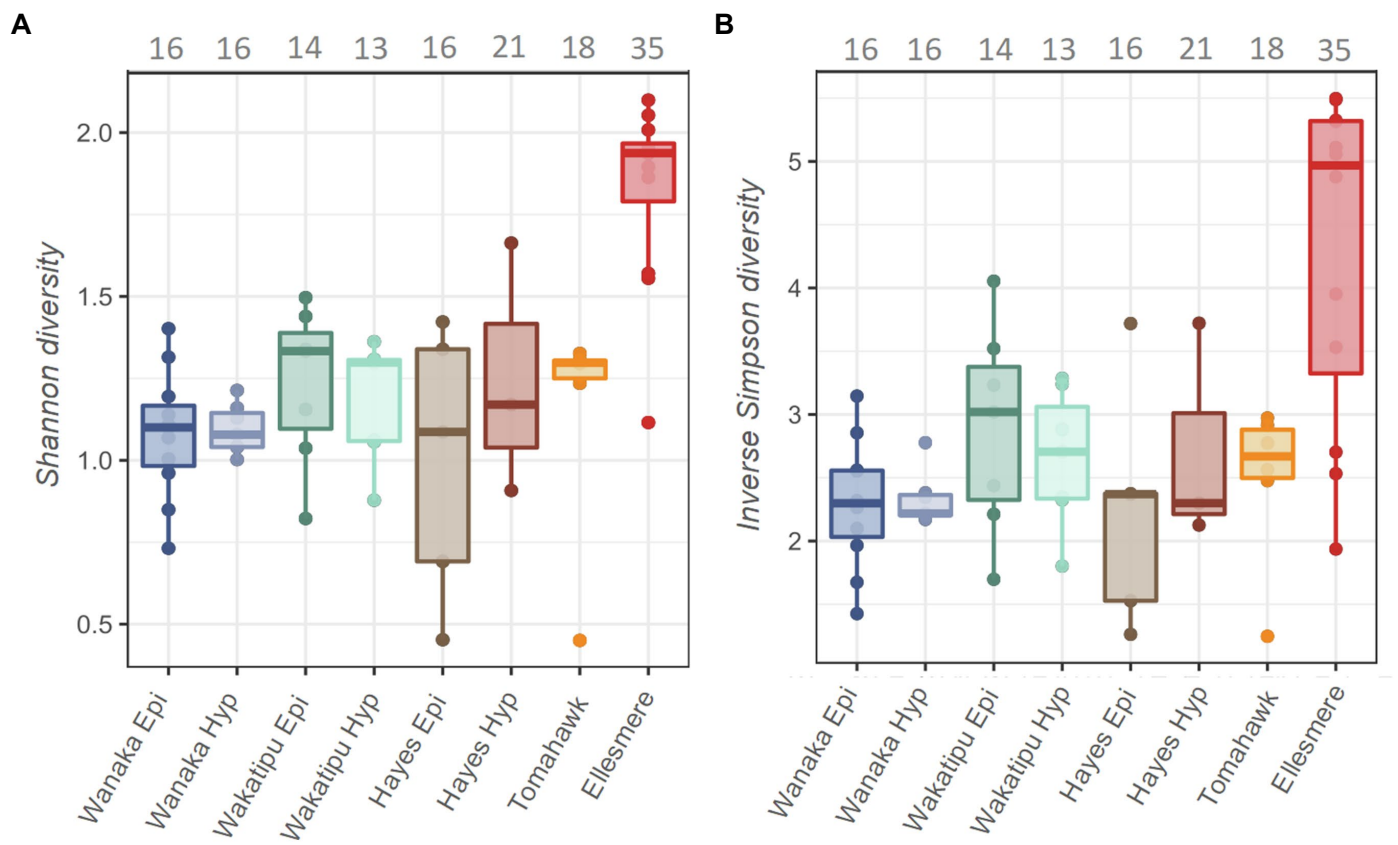
Shannon **(A)** and Inverse Simpson **(B)** alpha diversity estimates for the five study lakes, including samples from February 2018 to February 2019. Grey numbers above the plots indicate the total number of picocyanobacterial amplicon sequence variants (ASVs) in each lake over the 12-month period, prior to filtering and normalisation.

A total of 13,996 ASVs were recovered across the five study lakes, 5,946 (42%) of which were cyanobacterial (class: Oxyphotobacteria). After removal of chloroplasts, filtering, and sample rarefaction, 206 cyanobacterial ASVs remained of which 68 ASVs (33%) were picocyanobacterial (order: *Synechococcales*). The number of ASVs recovered at each processing step can be found in Supplementary Table S4. All abundant Pcy (>1% of total Pcy reads per sample) were taxonomically assigned to the genus *Cyanobium* PCC 6307 and resulting ASVs were numbered (e.g., CY.1) for analysis. Alpha diversity was significantly different between the lakes [ANOVA: *F*(6,44)=11.18, *p*<0.001] highest in Lake Ellesmere/Te Waihora (average Shannon diversity score=1.8; average Inverse Simpson score=4.3; Figure 3). Lakes Wanaka, Wakatipu, and Hayes returned the lowest alpha diversity scores, regardless of the weighting imposed by the differing methods (Figure 3). Temporal shifts in Pcy observed richness occurred within each lake, with Lake Ellesmere/Te Waihora and Tomahawk lagoon exhibiting a decrease in richness over the sampling period (Supplementary Figure S4). Richness remained relatively stable in the other lakes except for the Lake Hayes hypolimnion where there was a large increase in richness during January and February 2019 (Supplementary Figure S4).

**Figure 4:**
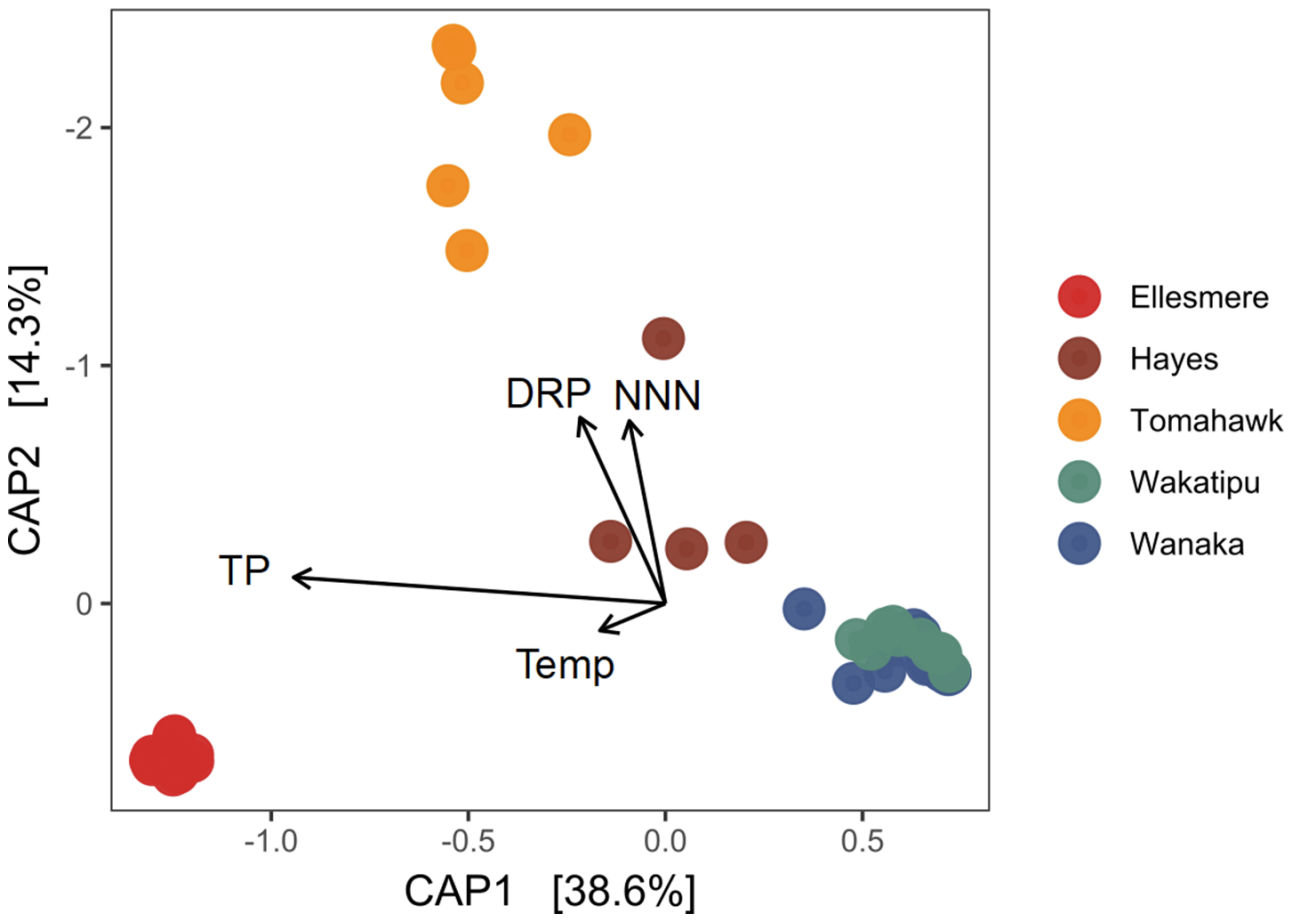
Canonical analysis of principal coordinates (CAP) ordination of picocyanobacterial communities found in the five study lakes over 1year of monthly sampling (February 2018 to February 2019) using a weighted Jaccard dissimilarity matrix. Constraints are significant (*p*<0.001). TP, total phosphorus; DRP, dissolved reactive phosphorus; Temp, temperature; and NNN, nitrate-nitrite nitrogen.

After filtering of potentially erroneous low-abundance ASVs and sample rarefaction, 68 Pcy ASVs were found across the five study lakes. Pcy communities were significantly different between the lakes using a weighted Jaccard dissimilarity matrix (PerMANOVA *F*=31.51, *R*^2^=0.73, *p*<0.001; Figure 4). However, variances were not homogeneous between lakes (*F*=2.68 *p*=0.039), meaning within-lake community differences could in-part be driving this significant result. Nevertheless, CAP showed that samples from individual lakes clearly clustered together except for Lakes Wanaka and Wakatipu where communities showed some overlap (Figure 4). These patterns were consistent using dissimilarity matrices incorporating both the presence and absence of ASVs (binary Jaccard) and read abundance (weighted Jaccard; Supplementary Figure S5). Environmental conditions varied between lakes, as expected given the gradient of trophic states sampled (Supplementary Figure S6). Of note were the low DO concentrations recorded in the hypolimnion of Lake Hayes.

The constrained variables explained 59% of variation in community dissimilarity and a PerMANOVA of the model found the constraints to be highly significant (*F*=14.70, *p*<0.001) along with the individual terms DRP, NNN, and TP (*p*=0.005 for each variable). CAP1 which explains 38.6% of the variation is related to trophic state, with TP and the removed collinear variables TN, Secchi depth and Chl-*a* associating strongly with that axis.

### Temporal and Spatial Drivers of Picocyanobacterial Communities in Contrasting Lakes

#### Oligotrophic Lakes

**Figure 5:**
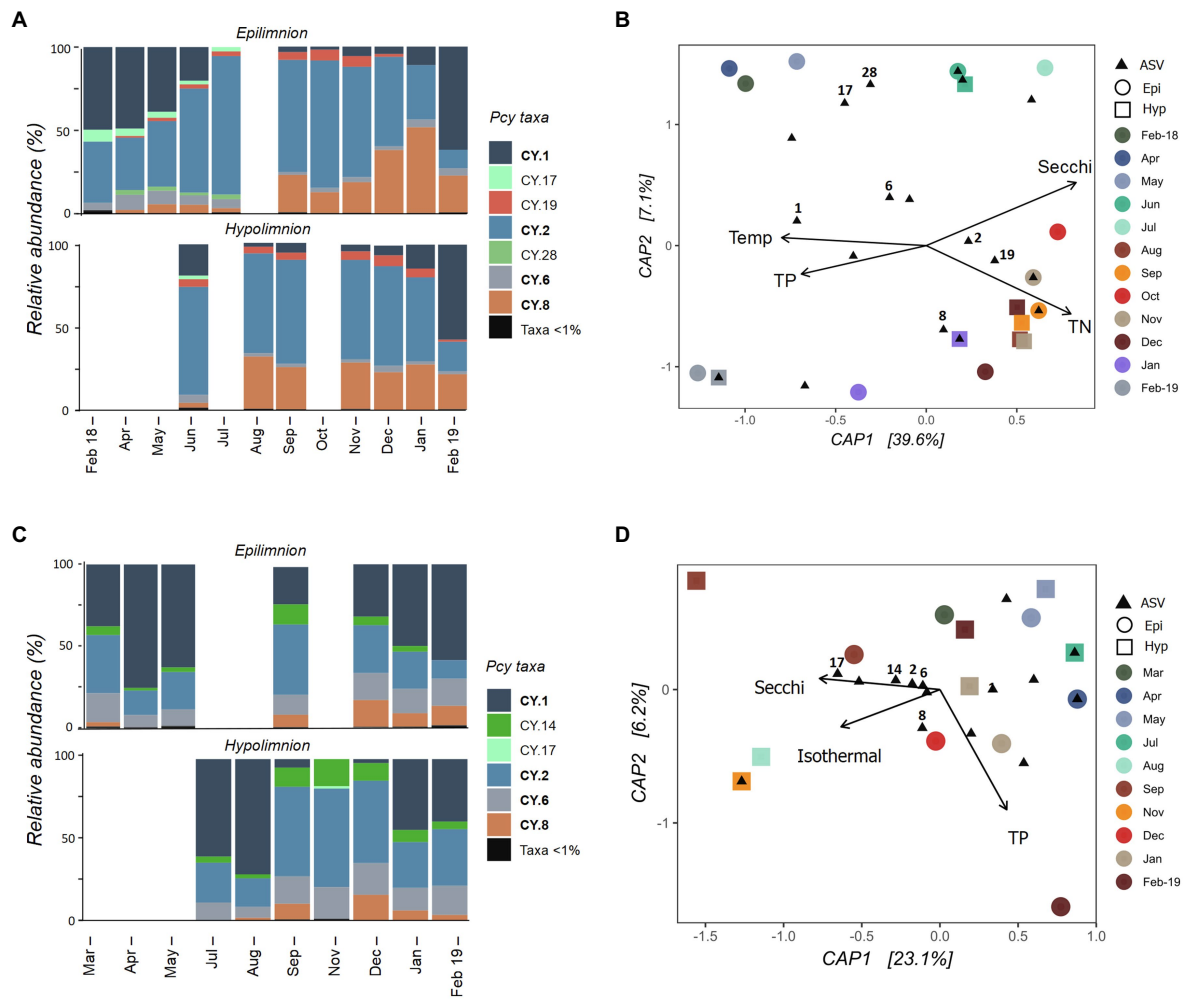
Picocyanobacterial communities in Lake Wanaka **(A,B)** and Wakatipu **(C,D)** from February 2018 to February 2019. **(A,C)** show relative abundances of dominant picocyanobacterial amplicon sequence variants (ASVs) in the epi- and hypolimnion throughout the year with rare ASVs (>1% read abundance per sample) grouped together. Taxa labelled in bold indicate ASVs that are shared in at least one other study lake. Blank bars are due to insufficient cyanobacterial reads. **(B,D)** are canonical analyses of principal coordinates (CAP) with picocyanobacterial sample communities (circles are epilimnion samples, squares are hypolimnion samples) and ASVs (black triangles), constrained by selected environmental variables. Abundant ASVs (>1% abundant per sample) are numbered. Pcy, picocyanobacteria; TN, total nitrogen; and TP, total phosphorus.

The Pcy community varied temporally in Lake Wanaka, with shifts in both the relative abundance of ASVs (Figure 5A) and the presence and absence of abundant ASVs throughout the year. Five hypolimnion samples and a single epilimnion sample produced insufficient reads and were removed after rarefaction (Figure 5A). Community composition and structure was highly similar in the epi- and hypolimnion from June 2018 to February 2019 when comparable samples were available, except for ASV CY.19 which remained abundant in the hypolimnion for 2months (January and February 2019) after becoming rare (<1% of sample reads) in the epilimnion (Figure 5A). A general decrease in the relative abundance of CY.1 and increase in CY.2 is seen in Winter and Spring (after lake turnover) in the epilimnion. In total, the environmental constraints explained 42% of the variation in the communities (Figure 5B). A PerMANOVA conducted on the constrained model showed that the constraints were significant (*F*=3.04, *p*=0.004), the horizontal axis CAP1 was significant (*F*=4.81, *p*=0.010), and Secchi depth was a highly significant constraint (*F*=3.23, *p*=0.010), followed by TP (*F*=2.43, *p*=0.025).

In oligotrophic Lake Wakatipu, six samples (three from each the epilimnion and hypolimnion) were removed during rarefaction. Remaining samples showed variation in both community composition and structure across the study months (Figure 5C). Relative abundances of ASVs CY.1 and CY.2 appear to shift in opposing ways, with lower relative abundances of CY.1 after lake turnover, particularly in the hypolimnion. Community composition was otherwise similar in the epi- and hypolimnion over the months sampled, with temporal variations in structure seen across both layers, appearing to correspond with lake turnover in August (Figure 5C; Supplementary Figure S3).

A constrained ordination using weighted Jaccard dissimilarities showed that environmental constraints explained 31% of the variation in Pcy communities (Figure 5D). However, PerMANOVA on the constrained model found the constraints to be insignificant in explaining community dissimilarities. In addition, most abundant ASVs cluster together near the origin of the CAP plot, further confirming that communities are similar across samples and the constraints are not sufficient at capturing the potential small amounts of variation observed.

#### Eutrophic Lakes

**Figure 6:**
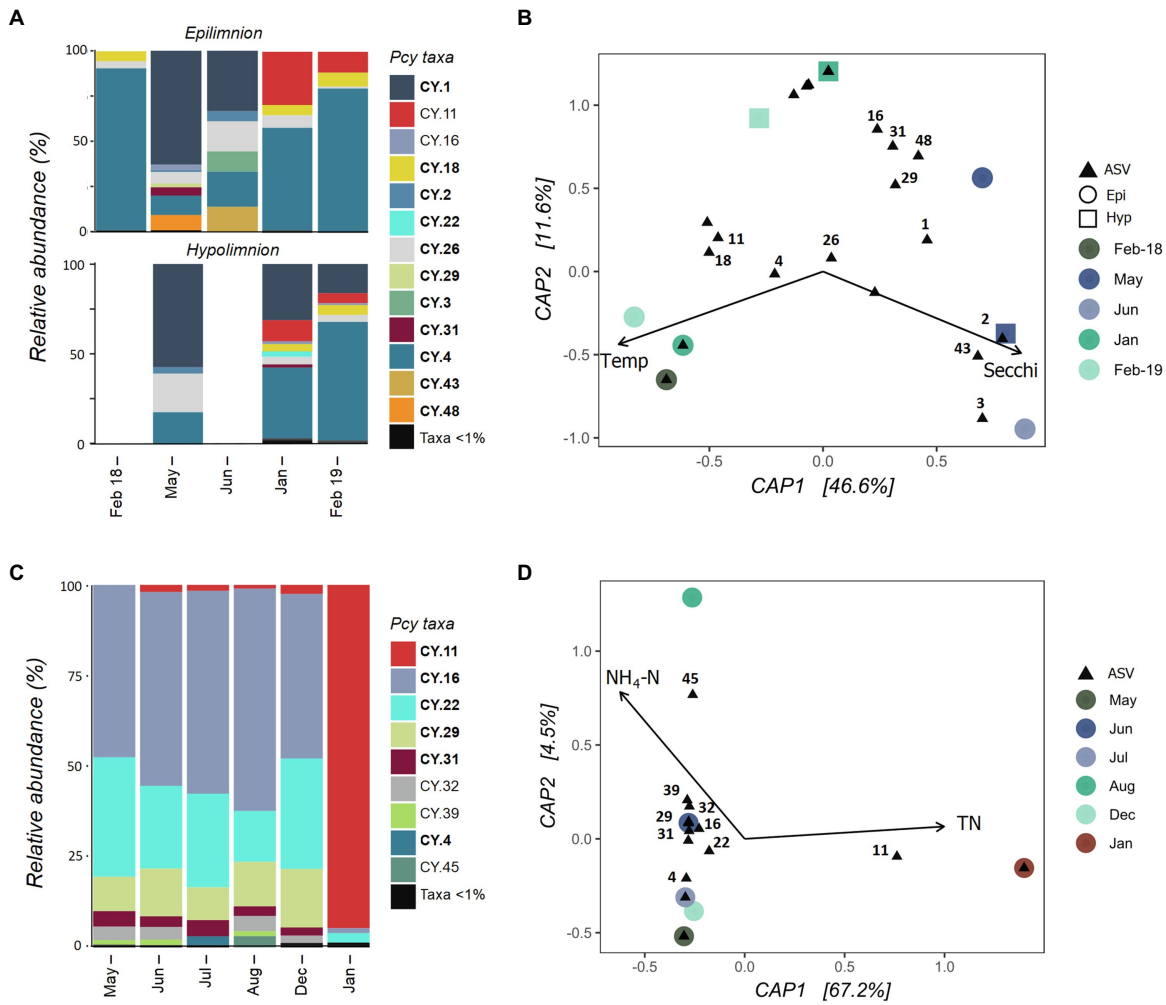
Picocyanobacterial communities in Lake Hayes **(A,B)** and Tomahawk Lagoon **(C,D)** from February 2018 to February 2019. **(A,C)** show relative abundances of dominant picocyanobacterial amplicon sequence variants (ASVs) throughout the year with rare ASVs (>1% read abundance per sample) grouped together. Taxa labelled in bold indicate ASVs that are shared in at least one other study lake. Blank bars are due to insufficient cyanobacterial reads. **(B,D)** are canonical analyses of principal coordinates (CAP) with picocyanobacterial sample communities (circles are epilimnion samples, squares are hypolimnion samples) and ASVs (black triangles), constrained by selected environmental variables. Abundant ASVs (>1% abundant per sample) are numbered. Pcy, picocyanobacteria; TN, total nitrogen; and NH_4_-N, ammoniacal nitrogen.

Collected samples from July 2018 to January 2019 in both the epi- and hypolimnion of Lake Hayes did not contain sufficient cyanobacterial reads and were removed after rarefaction along with the June hypolimnion sample. The resulting samples showed that the Pcy community in Lake Hayes was highly dynamic. The Pcy community in Autumn (May and June) was dominated by CY.1 with a shift to a CY.2 dominated community in the Summer (January and February; Figure 6A). Annual differences occurred in the epilimnion with CY.11 relatively abundant in January and February 2019 yet absent in February 2018. Epilimnetic communities also differ between May and June with CY.16, CY.31 and CY.48 abundant in May while in June CY.3 and CY.43 were abundant (Figure 6A). A PerMANOVA on the model found the constraints to be significant in explaining the community variation (*F*=3.48 and *p*=0.008), with Secchi depth highly significant (*F*=4.57 and *p*=0.005). Higher Secchi depths were related to the Autumn community, along with individual ASVs CY.2, CY.3, and CY.43, while CY.11, CY.18, and CY.4 were related to higher temperatures and CY.1 was related to lower temperatures (Figure 6B). However, Secchi depth was positively significantly correlated with TP and NH_4_-N, and temperature was positively significantly correlated with DO and negatively with DRP, suggesting that these variables may also be involved in the resulting community structure.

Tomahawk Lagoon was not sampled in February and November 2018 or February 2019. Samples from March, April, and September to November 2018 had insufficient cyanobacterial reads and were removed from the analysis post-rarefaction. The Pcy community was relatively similar in Tomahawk Lagoon over the months when Pcy were present, with the exception of January 2019 when the ASV CY.11 became highly abundant (Figure 6C). This sampling coincided with a large bloom of *Aphanizomenon* sp. and *Dolichospermum* sp. (Supplementary Figure S7), which together accounted for 96% of cyanobacterial reads. Lower-abundance ASVs (accounting for <10% of total reads) were variable throughout the year, with increases of CY.4 in July and CY.45 in August. The environmental constraints explained 67% of the variation (Figure 6D), however there were too few samples to confidently run a permutational analysis of the model. Nevertheless, both the bar plots and CAP suggest that the community in January 2019 diverges from other sampled months, correlated with high TN and associated with a large increase in the relative abundance of ASV CY.11. Interestingly, the increased abundance of CY.11 in Summer 2019 is seen in both eutrophic lakes.

**Figure 7:**
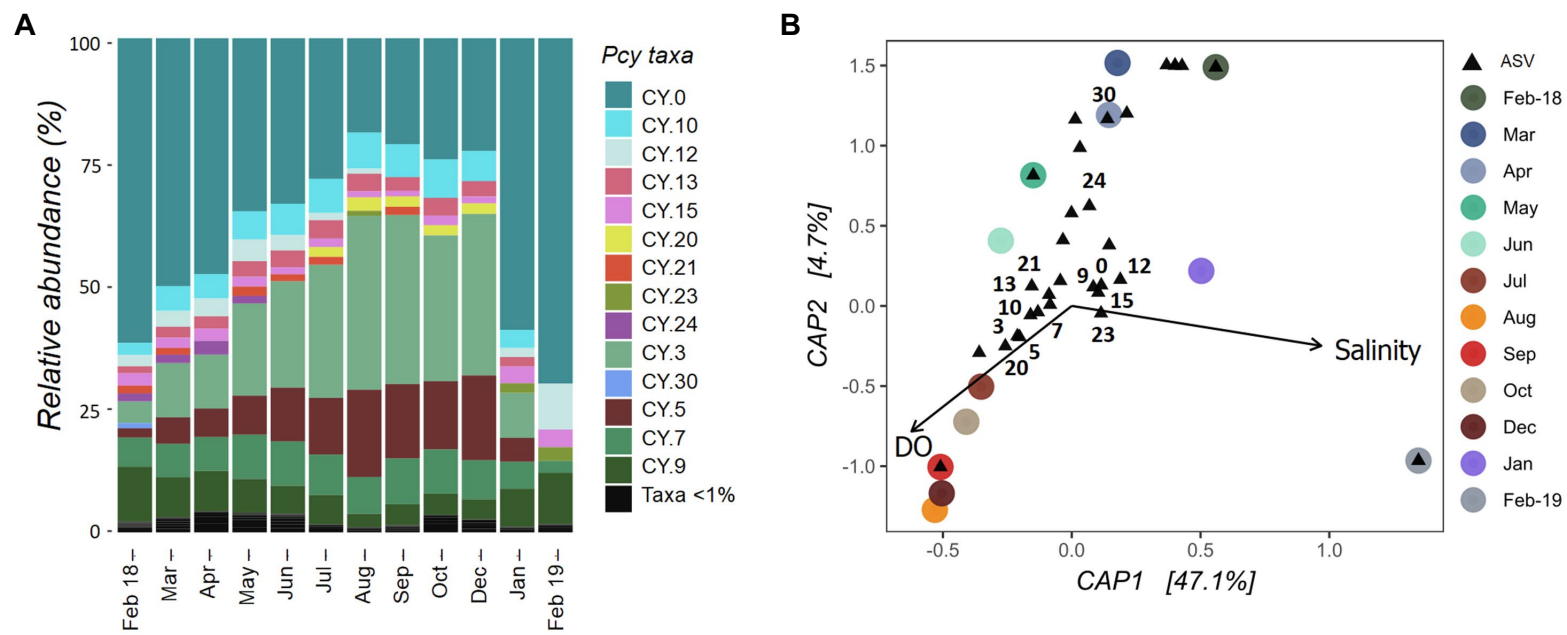
Lake Ellesmere/Te Waihora picocyanobacterial community from February 2018 to February 2019. **(A)** Relative abundance of picocyanobacterial amplicon sequence variants (ASVs) with rare ASVs (<1% total abundance per sample) grouped together. **(B)** canonical analyses of principal coordinates (CAP) ordination showing picocyanobacterial communities sampled each month (circles) with ASVs (black triangles), constrained by selected environmental variables. Abundant ASVs as shown in **(A)** are numbered **(B)**. Pcy, picocyanobacteria and DO, dissolved oxygen.

The Pcy community in Lake Ellesmere/Te Waihora showed temporal succession between February 2018 and December 2019 with a gradual increase in CY.3 and CY.5 (Figure 7A). Simultaneously, CY.0 and CY.12 decreased in relative abundance. An abrupt community shift occurred between December 2018 and January 2019 correlating with a lagoon opening event that took place from 12 December 2018 until 25 January 2019. DO and salinity explained 51% of the variation in community dissimilarities and were found to be significant (*F*=4.67 and *p*=0.001; Figure 7B). Despite three smaller lagoon opening events over the year, salinities remained between 5 and 8ppt from February 2018 to December 2018 before rising to 13.5ppt in February 2019. Subsequently, the community became heavily dominated by CY.0, with CY.12 and CY.9 also contributing high relative abundances. DO levels were highest from July to December 2018 (>100%), associated with ASVs CY.3, CY.5, CY.7, CY.10. CY.13, CY.20, and CY.21. ASVs CY.24 and CY.30 were abundant in February to April 2018, associated with lower DO levels.

### Vertical Picocyanobacterial Dynamics in Monomictic Lakes

T-tests confirmed NNN (Wanaka: *t*=2.08, *p*=<0.01, Wakatipu: *t*=2.16, *p*=>0.01) and TN (Wanaka: *t*=2.12, *p*=0.01, Wakatipu: *t*=2.08, *p*=>0.01) differed significantly between the epi- and hypolimnion of Lakes Wanaka and Wakatipu during the sampling period while other nutrients were similar with depth. Similarly, PAR differed between sampled depths in both lakes, with 1% surface PAR being reached between 22 and 39m depths in both lakes over the course of the year. However, PerMANOVA analysis (factor=depth) confirmed communities were not significantly different between the two vertical layers in either lake (Wanaka: *F*=1.07 and *p*=0.40, Wakatipu: *F*=1.44 and *p*=0.11).

Nutrient concentrations between the epi- and hypolimnion in eutrophic Lake Hayes were significantly different over the sampling period (DRP: *t*=2.08, *p*=<0.01, TP: *t*=2.12, *p*=0.04, and NH_4_-N: *t*=2.09, *p*=0.02). While a permutational test could not be performed due to the low number of samples, interesting differences in community structure and composition were observed between comparable epi- and hypolimnion samples (Figure 6A). For example, in May, 1month prior to lake turnover, the epilimnion contained ASVs (CY.16, CY.29, CY.31, and CY.48) not present in the hypolimnion sample. Conversely, during strong stratification in January and February 2019, CY.22 and CY.31 were abundant in the hypolimnion yet not present in the epilimnion (Figure 6A). During January and February 2019, DO at the hypolimnion sampling depth was <1.5%, indicating that these ASVs may be able to tolerate severe hypoxia.

## Discussion

### Temporal Abundances and Environmental Correlations

Picocyanobacterial abundances differed between and within lakes, with supertrophic Lake Ellesmere/Te Waihora consistently returning the highest cell abundances. In general, abundances in the oligotrophic lakes were less variable compared to the eutrophic lakes. Trophic state appeared to be related to pigment type and overall temporal patterns of abundance. PE-rich cells were dominant in oligotrophic lakes while eutrophic lakes contained both PE and PC cells at differing ratios and supertrophic Lake Ellesmere/Te Waihora contained only PC cells. Previous studies support these findings (Vörös et al., 1998; Vila and Abella, 2001), however eutrophic lakes have typically been found to contain predominantly PC cells, whereas the present study found eutrophic Lake Hayes to be dominated by PE cells. Lake Hayes is a complex lake known to fluctuate between clear and turbid water states and is showing signs of slowly recovering from eutrophication (Schallenberg, 2020). This variable water quality with periods of high water clarity may explain the higher than predicted proportion of PE-rich cells found.

#### Single Cell Abundances and Environmental Correlations

Studies have found that single or bimodal abundance peaks occurring in Spring or early Summer and Summer-Autumn are common for temperate oligotrophic lakes while abundances in eutrophic lakes tend to peak during Summer (Callieri et al., 2012b). The present study supports this, with a single Spring peak and bimodal Autumn and Winter peaks recorded in the epilimnion of oligotrophic lakes Wanaka and Wakatipu, respectively. In these lakes, Pcy abundances in the hypolimnion were related to lake turnover with abundances increasing significantly once the lakes became isothermal. In Winter, cell abundances were higher in the hypolimnion than the epilimnion in Lake Wanaka. Interestingly, NNN was negatively correlated with total cell abundances in the hypolimnion of Lakes Wanaka and Wakatipu, while TP was negatively correlated with Wakatipu epilimnion abundances. Negative responses of Pcy to nutrient addition (N and P) have been previously reported in oligotrophic lakes and mesocosms (Rhew et al., 1999; Burns and Schallenberg, 2001; Schallenberg and Burns, 2001; Burns and Galbraith, 2007; Yang et al., 2019), however the reasons for this response remain unknown and require further study.

Total cell abundances in the eutrophic and supertrophic lakes were not significantly correlated with any measured environmental variables. In the small, eutrophic lakes (Hayes and Tomahawk), strong Summer peaks were followed by low abundances and sometimes absences of Pcy in other seasons and months, a common pattern that has been reported from other eutrophic systems (Callieri et al., 2012b). Conversely, supertrophic Lake Ellesmere/Te Waihora had consistently high abundances throughout the year. While it is generally accepted that Pcy may reach high abundances in eutrophic lakes, their relative importance and contribution to total phytoplankton biomass is thought to decrease with increasing trophic state (Stockner, 1991; Bell and Kalff, 2001). Often, cell abundances decrease with increasing trophic state, accompanied by an increase in picoeukaryotes (Ruber et al., 2018). Pick (2000) showed that the relationship between cell abundances and TP or trophic state is not necessarily linear, instead following a polynomial function whereby Pcy abundances increase linearly up to TP concentrations of ~15μgL^−1^ before declining. However, some supertrophic lakes and coastal lagoons such as Lake Ellesmere/Te Waihora (average TP over the study period=200μgL^−1^) do not appear to adhere to this model, with abundances often high and particularly difficult to predict (Bec et al., 2011; Callieri et al., 2012b; Caroppo, 2015). Microbial studies in brackish, eutrophic lakes are increasingly revealing significant Pcy populations which may have been previously overlooked (Carrick and Schelske, 1997; Pulina et al., 2017; Schallenberg et al., 2021), demonstrating the need to improve our knowledge of microbial foodwebs in these systems. This is particularly pertinent with sea level rise and climate change predicted to significantly affect these habitats (Carrasco et al., 2016).

#### Colonies and Environmental Correlations

The relatively low number of colonial cells found in the oligotrophic lakes is not surprising given that eutrophic environments tend to favour these morphotypes (Callieri et al., 2012b). However, high numbers of colonies were recorded in certain months, e.g., Lake Wanaka in February 2019 >50% of cells formed colonies. Many hypotheses exist around the drivers of colony formation in temperate oligotrophic lakes including possible ease of nutrient transfer in times of nutrient depletion, UV protection and predator avoidance (Klut and Stockner, 1991; Crosbie et al., 2003; Callieri et al., 2016). Colonial peaks in the epilimnion occurred in Summer in both oligotrophic lakes when UV levels are typically highest. The February 2019 colonial peak in Lake Wanaka coincided with a 50% drop in NNN concentrations, while colonial cells in the hypolimnion of Lake Wakatipu were significantly negatively correlated with NNN. Interestingly, no colonies were found in eutrophic Lake Hayes during this study. In contrast, colonies in eutrophic Tomahawk Lagoon and supertrophic Lake Ellesmere/Te Waihora were common, accounting for up to 100% of Pcy cells suggesting that colonial morphotypes are commonly associated with brackish eutrophic coastal lakes and lagoons. Colonial cells in Tomahawk lagoon and Lake Ellesmere/Te Waihora were positively correlated with certain nutrients. This contrasts with the negative relationship found in Lake Wakatipu, indicating that Pcy in these lakes respond differently to drivers relating to trophic state. The abundance of colonial cells in Lake Ellesmere/Te Waihora dropped drastically in February 2019, 2months after the lake was opened to the sea, implying that significant mixing with the marine environment may have reduced colonial morphotypes.

In line with our first hypothesis, cell abundances were shown to be temporally dynamic within the study lakes. This was also the case in both the epi- and hypolimnion of monomictic lakes. While cell abundances were correlated with environmental variables, responses were inconsistent between lakes and no environmental variables were significantly correlated with abundances across all lakes, or within trophic states.

### Diversity and Dynamics of Picocyanobacterial Communities Between Lakes

Pcy communities were diverse in all study lakes and these communities differed significantly between most lakes, confirming our second hypothesis. Shannon diversity differed significantly between the lakes and overall patterns of diversity were consistent using both the Shannon and Inverse Simpson alpha diversity indices which are weighted towards rare and abundant species, respectively. Observed richness was highest in supertrophic lake Ellesmere/Te Waihora and varied temporally in the supertrophic and eutrophic lakes.

The five lakes revealed diverse and distinct Pcy communities, except for oligotrophic Lakes Wanaka and Wakatipu which shared five abundant ASVs. These lakes are similar in trophic state, type, size and proximity (Table 2). However, the results of this study suggest that proximity is less important in determining Pcy community structure than local conditions and trophic state. For example, eutrophic Lake Hayes and oligotrophic Lake Wakatipu are situated with 15km of each other yet contain significantly different Pcy communities. The physico-chemical variables TP, NNN and DRP were identified as significant drivers differentiating Pcy communities among the five study lakes. These drivers along with their removed cross-correlated variables are related to lake trophic state, suggesting that trophic state may be a significant driver of Pcy community structure across lakes. The present findings contrast somewhat with those of Wood et al. (2017) who found several dominant *Synechococcus* OTU’s persisted in lakes across a range of trophic states. Similarly, Ruber et al. (2016) found that Pcy communities were similar in trophically contrasting, connected lakes. One possible reason for this could be the use of ASVs in the present study while Wood et al. (2017) and Ruber et al. (2016) used OTU clustering, potentially allowing for numerous ASVs to group as one OTU. The connectedness of the lakes in Ruber et al. (2016) could also explain the similarities found, suggesting that dispersal is stronger than local selection in connected lakes. It is also likely other factors not included in this study are responsible for the remaining unexplained variation and a larger dataset including community analysis in lakes across the full trophic gradient might reveal more detailed community patterns.

### Temporal Drivers of Picocyanobacterial Communities in Contrasting Lakes

Picocyanobacterial communities within lakes varied temporally and these shifts were often related to changing environmental conditions. Lakes Wanaka and Wakatipu communities revealed a seasonal succession of abundant ASVs which was related to Secchi depth and TP. Although the potential contribution of Chl-*a* and DRP in shaping these communities should not be ignored given these variables were removed due to significant negative and positive correlations with Secchi depth, respectively. Similarly in Lake Wakatipu, Secchi depth and TP were among the best predictors of community dissimilarities, however these were not statistically significant. ASVs shared between the two oligotrophic lakes were abundant at the same times of year except for CY.1 which peaked in relative abundance in Summer in Lake Wanaka while being a substantial component in Lake Wakatipu in Summer, Autumn, and Winter. Across both lakes, CY.1 and CY.2 alternate as being the most abundant ASV present. Phylogenetic analysis by Schallenberg et al. (2021) suggests that these ASVs are not closely related, belonging to Group A *Cyanobium gracile* and Clade III, respectively.

Temporal analysis of eutrophic Lake Hayes suggests a highly dynamic and responsive Pcy community, with large temporal shifts in the presence/absence and relative abundance of ASVs. These shifts occurred both between seasons and within seasons, related to Secchi depth and temperature. Lake Hayes is a eutrophic, monomictic lake that often experiences hypoxia in the epilimnion and anoxia in the hypolimnion over Summer (Bayer et al., 2008). While DO was not included in the CAP analysis due to multiple instances of collinearity with other variables, it was positively correlated with temperature suggesting that it is also related to community structuring. These results suggest niche specificity of certain Pcy ASVs in Lake Hayes as well as a rapid adaptation to changing environmental conditions. Pcy community structure in Tomahawk Lagoon was relatively stable between seasons until a major shift in Pcy dominance in January 2019. Within 1month, two previously abundant ASVs disappeared and one ASV became highly abundant. This shift was related to a bloom of *Dolichospermum* sp. and *Aphanizomenon* sp., suggesting that co-occurring cyanobacteria may cause strain-specific Pcy responses. Certain Pcy have been found associated with blooms of larger filamentous cyanobacteria (Pick, 2016) with both positive and negative relationships (Śliwińska-Wilczewska et al., 2018). Śliwińska-Wilczewska et al. (2017) found that *Synechococcus* sp. isolates had a positive effect on the growth of *Aphanizomenon flos*-*aquae*, whilst negatively affecting other filamentous species. The dramatic increase in CY.11 found in Tomahawk Lagoon during the filamentous cyanobacterial bloom could indicate a positive interaction with *Aphanizomenon* sp. or *Dolichospermum* sp.

The Lake Ellesmere/Te Waihora Pcy community underwent gradual temporal succession for most of the year followed by a large shift in community structure after the ICOLL was opened to the ocean in December 2018. The corresponding increase in salinity was reflected by a major shift in Pcy dominance, suggesting halotolerance of certain ASVs. Analysis of corresponding environmental drivers confirmed that these shifts were related to salinity and DO levels. Given the highly dynamic nature of ICOLLS including temporal shifts in water quality, salinity and water level (Schallenberg et al., 2010), Pcy in these systems are subject to dramatic environmental change. The dominance of Pcy in some hypertrophic lakes and lagoons (Sorokin and Dallocchio, 2008; Schapira et al., 2010; Bec et al., 2011; Albrecht et al., 2017; Pulina et al., 2017) might be explained by the high adaptability of freshwater Pcy (Callieri, 2017; Huber et al., 2017). The present results suggest that temporal succession of different ASVs could explain this adaptability, allowing Pcy to persist at high abundances while community composition varies in response to environmental change.

Of the potential physico-chemical variables included in this study none were consistent in driving communities across all study lakes. This is not surprising given the different trophic conditions and individual ASVs dominating the Pcy communities. In support of our third hypothesis, we have shown that within-lake communities are temporally dynamic, and abundant ASVs are associated with specific environmental drivers. Different species were shown to fill specific niches as environmental conditions changed. In addition, certain ASVs were found to persist at rare levels (<1% of sample Pcy reads) before significantly increasing in relative abundance within months. Teasing apart the many potential abiotic drivers of Pcy is difficult due to the cross-correlated nature of variables and the effects of seasonality (Callieri et al., 2012b). However, we found patterns of gradual succession and rapid disturbance following disruptive events such as mixing in deep lakes and marine influence in a coastal lagoon. It is likely that drivers other than those measured in this analysis may explain some of the variation in community structure. For example, Callieri et al. (2016) and Huber et al. (2017) found that grazing pressure by zooplankton can exert preferential pressure on certain Pcy phenotypes. Future studies would benefit from incorporating potential biotic drivers which may help explain Pcy abundances and community structure in freshwater systems.

### Picocyanobacteria Community Dynamics in Deep Stratified Lakes

Vertical partitioning of Pcy strains is common in the oligotrophic marine environment where significant temperature and light gradients create niches utilised by various clades of marine Pcy (Six et al., 2007; Scanlan et al., 2009; Bertos-Fortis et al., 2016; Farrant et al., 2016). Such depth partitioning was also found in Lake Maggiore where Pcy OTU’s differed with depth, particularly during Summer stratification (Callieri et al., 2012a) and high altitude Andean lakes (Caravati et al., 2010). However, no significant vertical partitioning of Pcy communities was found in monomictic, oligotrophic Lakes Wanaka and Wakatipu over the course of the year. While community structure varied temporally, this structure was mirrored in both the epi- and hypolimnion of these lakes. This contrasts with findings from Lake Maggiore, a similarly large peri-alpine, oligotrophic lake, however these results are similar to those found by Ruber et al. (2018) in the Osterseen lakes of Bavaria. There, relatively small differences in Pcy OTU’s were found with depth while temporal community shifts were greater (Ruber et al., 2018). While TN and NNN differed significantly with depth in these study lakes, along with PAR, it is possible that these differences are not strong enough to drive community shifts in these lakes. In the present study, a lack of comparable epi- and hypolimnetic samples during both isothermal and mixed periods, meant that that it was not possible to use a nested analysis of both depth and mixing phase to determine the influence of mixing phase on vertical communities.

In contrast to these large, monomictic, oligotrophic lakes, vertical partitioning of the Pcy community was strong in eutrophic, monomictic Lake Hayes although due to the limited sample size this could not be tested statistically. Physico-chemical conditions differed significantly between the epi-and hypolimnion over the study period. In all 3months with comparable epi- and hypolimnion samples, communities differed in both structure (presence/absence) and composition (relative abundance). Of particular interest was the presence of ASVs in the hypolimnion of Lake Hayes during Summer hypoxic conditions. This indicates that Pcy may not only survive under severe hypoxia, but certain ASVs that appear and become relatively abundant during this time may have a selective advantage under these conditions. While DNA metabarcoding alone cannot determine whether cells are alive and viable, survival under hypoxic and anoxic conditions may not be uncommon for Pcy, with the recent discovery of viable Pcy at 750m depth in the anoxic Black Sea (Callieri et al., 2019). The strains isolated from this depth had the ability to accumulate Chl-*a* under dark anoxic conditions and photosynthesise when exposed to oxic, light conditions (Callieri et al., 2019). This finding provides further evidence of picocyanobacterial ubiquity and challenges our understanding of Pcy physiology by highlighting the extreme environments inhabited by certain strains.

In contrast to our fourth hypothesis, these results indicate that vertical partitioning of Pcy communities in deep lakes is not always present, can be variable and may be lake-specific. We recommend the use of larger datasets to statistically confirm differences between stratified and mixed periods, along with culturing of strains of interest from both the epi- and hypolimnion of stratified lakes to assess optimal growth conditions. This is of particular interest in low-oxygen environments such as Lake Hayes, where differing photosynthetic and metabolic pathways could be used. The use of metagenomics and metatranscriptomics would provide further information on the functions of these strains and how their functional profiles shift with changing environments.

## Conclusion

The temporal and vertical dynamics of freshwater Pcy were explored in five lakes with contrasting trophic states using a combination of epifluorescence microscopy and environmental DNA metabarcoding. Abundances differed between and within lakes, and there were few relationships with environmental variables. However, these responses were not consistent across all lakes. The addition of community-scale analysis revealed Pcy communities were diverse and differed significantly between lakes. Pcy communities within the lakes were temporally dynamic and certain ASVs were strongly associated with lake-specific environmental drivers. Shifts in community composition were often related to environmental changes, indicating that lacustrine Pcy respond rapidly to change through adapting community structure. This study shows that community-scale responses allow Pcy to persist at high abundances through seasonal changes and disruptive events including isothermal mixing in deep lakes and a marine intrusion in a coastal lagoon. Finally, vertical structuring of Pcy communities was found only in one eutrophic lake, suggesting vertical niche partitioning may not be as common in lakes as in the marine environment. Together these results demonstrate that a combination of microscopy and DNA metabarcoding can lead to an improved understanding of freshwater Pcy dynamics and reveal strain-specific drivers. We recommend that future studies cease referring to Pcy as one functional group and utilise molecular techniques to allow for the identification of individual genotypes and their specific drivers.

## Data Availability Statement

The datasets presented in this study can be found in online repositories. The names of the repository/repositories and accession number(s) can be found at: https://www.ncbi.nlm.nih.gov/bioproject/749894. Bioinformatic and statistical analysis scripts can be found at: https://github.com/lenaschall/Temporal_Picocyanobacteria_Scripts.

## Author Contributions

LS, SW, and CB conceived and designed the study. LS analyzed the data, prepared figures and tables, and authored the manuscript draft. JP and SW helped with metabarcoding and statistical analysis. All authors contributed to the article and approved the submitted version.

## Funding

This research was funded by the New Zealand Ministry of Business, Innovation and Employment research programme, “Enhancing the health and resilience of New Zealand lakes” (UOWX1503) and Our lakes’ health; past, present, and future (C05X1707). LS thanks the University of Otago for a Ph.D scholarship.

## Conflict of Interest

The authors declare that the research was conducted in the absence of any commercial or financial relationships that could be construed as a potential conflict of interest.

## Publisher’s Note

All claims expressed in this article are solely those of the authors and do not necessarily represent those of their affiliated organizations, or those of the publisher, the editors and the reviewers. Any product that may be evaluated in this article, or claim that may be made by its manufacturer, is not guaranteed or endorsed by the publisher.

## References

[ref1] Albrecht M. Pröschold T. Schumann R. (2017). Identification of cyanobacteria in a eutrophic coastal lagoon on the southern Baltic coast. Front. Microbiol. 8:923. doi: 10.3389/fmicb.2017.00923, PMID: 28611738 PMC5446986

[ref2] Bayer T. K. (2013). Effects of Climate Change on Two Large, Deep Oligotrophic Lakes in New Zealand. dissertation/master’s thesis. Dunedin: University of Otago.

[ref3] Bayer T. K. Schallenberg M. Martin C. E. (2008). Investigation of nutrient limitation status and nutrient pathways in Lake Hayes, Otago, New Zealand: a case study for integrated lake assessment. N. Z. J. Mar. Freshw. Res. 42, 285–295. doi: 10.1080/00288330809509956

[ref4] Bec B. Collos Y. Souchu P. Vaquer A. Lautier J. Fiandrino A.. (2011). Distribution of picophytoplankton and nanophytoplankton along an anthropogenic eutrophication gradient in French Mediterranean coastal lagoons. Aquat. Microb. Ecol. 63, 29–45. doi: 10.3354/ame01480

[ref5] Bell T. Kalff J. (2001). The contribution of picophytoplankton in marine and freshwater systems of different trophic status and depth. Limnol. Oceanogr. 46, 1243–1248. doi: 10.4319/lo.2001.46.5.1243

[ref6] Bertos-Fortis M. Farnelid H. M. Lindh M. V. Casini M. Andersson A. Pinhassi J.. (2016). Unscrambling cyanobacteria community dynamics related to environmental factors. Front. Microbiol. 7:625. doi: 10.3389/fmicb.2016.00625, PMID: 27242679 PMC4860504

[ref7] Burns C. W. Galbraith L. M. (2007). Relating planktonic microbial food web structure in lentic freshwater ecosystems to water quality and land use. J. Plankton Res. 29, 127–139. doi: 10.1093/plankt/fbm001

[ref8] Burns C. W. Schallenberg M. (2001). Short-term impacts of nutrients, *daphnia* and copepods on microbial food-webs of an oligotrophic and eutrophic lake. N. Z. J. Mar. Freshw. Res. 35, 695–710. doi: 10.1080/00288330.2001.9517036

[ref9] Burns C. W. Stockner J. G. (1991). Picoplankton in six New Zealand lakes: abundance in relation to season and trophic state. Int. Rev. Gesamten Hydrobiol. Hydrogr. 76, 523–536. doi: 10.1002/iroh.19910760405

[ref10] Cabello-Yeves P. J. Picazo A. Camacho A. Callieri C. Rosselli R. Roda-Garcia J. J.. (2018). Ecological and genomic features of two widespread freshwater picocyanobacteria. Environ. Microbiol. 20, 3757–3771. doi: 10.1111/1462-2920.14377, PMID: 30117250

[ref11] Cai Y. Kong F. (2013). Diversity and dynamics of picocyanobacteria and the bloom-forming cyanobacteria in a large shallow eutrophic lake (Lake Chaohu, China). J. Limnol. 72:38. doi: 10.4081/jlimnol.2013.e38

[ref12] Callahan B. J. McMurdie P. J. Rosen M. J. Han A. W. Johnson A. J. A. Holmes S. P. (2016). DADA2: high-resolution sample inference from Illumina amplicon data. Nat. Methods 13, 581–583. doi: 10.1038/nmeth.3869, PMID: 27214047 PMC4927377

[ref15] Callieri C. (2017). *Synechococcus* plasticity under environmental changes. FEMS Microbiol. Lett. 364, 1–8. doi: 10.1093/femsle/fnx229, PMID: 29092031

[ref16] Callieri C. Amalfitano S. Corno G. Bertoni R. (2016). Grazing-induced *Synechococcus* microcolony formation: experimental insights from two freshwater phylotypes. FEMS Microbiol. Ecol. 92:fiw154. doi: 10.1093/femsec/fiw154, PMID: 27411979

[ref17] Callieri C. Caravati E. Corno G. Bertoni R. (2012a). Picocyanobacterial community structure and space-time dynamics in the subalpine Lake Maggiore (N. Italy). J. Limnol. 71:9. doi: 10.4081/jlimnol.2012.e9

[ref19] Callieri C. Cronberg G. Stockner J. G. (2012b). “Freshwater picocyanobacteria- single cells, microcolonies and colonial forms.” in Ecol Cyanobacteria II Their Divers Space Time. ed. Whitton B. (Springer, Netherlands: Dordrecht), 229–269.

[ref20] Callieri C. Slabakova V. Dzhembekova N. Slabakova N. Peneva E. Cabello-Yeves P. J.. (2019). The mesopelagic anoxic Black Sea as an unexpected habitat for *Synechococcus* challenges our understanding of global “deep red fluorescence”. ISME J. 13, 1676–1687. doi: 10.1038/s41396-019-0378-z, PMID: 30820035 PMC6776005

[ref21] Callieri C. Stockner J. G. (2002). Freshwater autotrophic picoplankton: a review. J. Limnol. 61:1. doi: 10.4081/jlimnol.2002.1

[ref22] Caravati E. Callieri C. Modenutti B. Corno G. Balseiro E. Bertoni R.. (2010). Picocyanobacterial assemblages in ultraoligotrophic Andean lakes reveal high regional microdiversity. J. Plankton Res. 32, 357–366. doi: 10.1093/plankt/fbp126

[ref23] Caroppo C. (2015). Ecology and biodiversity of picoplanktonic cyanobacteria in coastal and brackish environments. Biodivers. Conserv. 24, 949–971. doi: 10.1007/s10531-015-0891-y

[ref24] Carrasco A. R. Ferreira Ó. Roelvink D. (2016). Coastal lagoons and rising sea level: a review. Earth-Sci. Rev. 154, 356–368. doi: 10.1016/j.earscirev.2015.11.007

[ref25] Carrick H. J. Schelske C. L. (1997). Have we overlooked the importance of small phytoplankton in productive waters? Limnol. Oceanogr. 42, 1613–1621. doi: 10.4319/lo.1997.42.7.1613

[ref26] Crosbie N. Teubner K. Weisse T. (2003). Flow-cytometric mapping provides novel insights into the seasonal and vertical distributions of freshwater autotrophic picoplankton. Aquat. Microb. Ecol. 33, 53–66. doi: 10.3354/ame033053

[ref27] Di Cesare A. Dzhembekova N. Cabello-Yeves P. J. Eckert E. M. Slabakova V. Slabakova N.. (2020). Genomic comparison and spatial distribution of different *Synechococcus* phylotypes in the Black Sea. Front. Microbiol. 11:1979. doi: 10.3389/fmicb.2020.01979, PMID: 32903389 PMC7434838

[ref28] Dormann C. F. Elith J. Bacher S. Buchmann C. Carl G. Carré G.. (2013). Collinearity: a review of methods to deal with it and a simulation study evaluating their performance. Ecography 36, 27–46. doi: 10.1111/j.1600-0587.2012.07348.x

[ref29] Farrant G. K. Doré H. Cornejo-Castillo F. M. Partensky F. Ratin M. Ostrowski M.. (2016). Delineating ecologically significant taxonomic units from global patterns of marine picocyanobacteria. Proc. Natl. Acad. Sci. 113, E3365–E3374. doi: 10.1073/pnas.1524865113, PMID: 27302952 PMC4914166

[ref30] Felföldi T. Duleba M. Somogyi B. Vajna B. Nikolausz M. Présing M.. (2011). Diversity and seasonal dynamics of the photoautotrophic picoplankton in Lake Balaton (Hungary). Aquat. Microb. Ecol. 63, 273–287. doi: 10.3354/ame01501

[ref31] Harke M. J. Steffen M. M. Gobler C. J. Otten T. G. Wilhelm S. W. Wood S. A.. (2016). A review of the global ecology, genomics, and biogeography of the toxic cyanobacterium, *Microcystis* spp. Harmful Algae 54, 4–20. doi: 10.1016/j.hal.2015.12.007, PMID: 28073480

[ref32] Huber P. Diovisalvi N. Ferraro M. Metz S. Lagomarsino L. Llames M. E.. (2017). Phenotypic plasticity in freshwater picocyanobacteria. Environ. Microbiol. 19, 1120–1133. doi: 10.1111/1462-2920.13638, PMID: 27943603

[ref33] Huisman J. Codd G. A. Paerl H. W. Ibelings B. W. Verspagen J. M. H. Visser P. M. (2018). Cyanobacterial blooms. Nat. Rev. Microbiol. 16, 471–483. doi: 10.1038/s41579-018-0040-1, PMID: 29946124

[ref34] Jasser I. Callieri C. (2017). “Analysis of picocyanobacteria abundance in epifluorescence microscopy” in Handb Cyanobacterial Monit Cyanotoxin Anal. eds. Meriluoto J. Spoof L. Codd G. A. (Chichester, UK: John Wiley & Sons, Ltd.), 339–342.

[ref35] Jasser I. Karnkowska-Ishikawa A. Kozłowska E. Królicka A. Łukomska-Kowalczyk M. (2010). Composition of picocyanobacteria community in the great Mazurian Lakes: isolation of phycoerythrin-rich and phycocyanin-rich ecotypes from the system – comparison of two methods. Pol. J. Microbiol. 59, 21–31. doi: 10.33073/pjm-2010-003, PMID: 20568526

[ref36] Johnson Z. I. Zinser E. R. Coe A. McNulty N. P. Woodward E. M. S. Chisholm S. W. (2006). Niche partitioning among *Prochlorococcus* ecotypes along ocean-scale environmental gradients. Science 311, 1737–1740. doi: 10.1126/science.1118052, PMID: 16556835

[ref37] Kandlikar G. S. Gold Z. J. Cowen M. C. Meyer R. S. Freise A. C. Kraft N. J. B.. (2018). Ranacapa: an R package and shiny web app to explore environmental DNA data with exploratory statistics and interactive visualizations. F1000Research 7:1734. doi: 10.12688/f1000research.16680.1, PMID: 30613396 PMC6305237

[ref38] Klut M. E. Stockner J. G. (1991). Picoplankton associations in an ultra-oligotrophic lake on Vancouver Island, British Columbia. Can. J. Fish. Aquat. Sci. 48, 1092–1099. doi: 10.1139/f91-129

[ref39] Lavallée B. Pick F. (2002). Picocyanobacteria abundance in relation to growth and loss rates in oligotrophic to mesotrophic lakes. Aquat. Microb. Ecol. 27, 37–46. doi: 10.3354/ame027037

[ref40] Martin M. (2011). Cutadapt removes adapter sequences from high-throughput sequencing reads. EMBnet.journal 17, 10–12. doi: 10.14806/ej.17.1.200

[ref41] McMurdie P. J. Holmes S. (2013). Phyloseq: an R package for reproducible interactive analysis and graphics of microbiome census data. PLoS One 8:e61217. doi: 10.1371/journal.pone.0061217, PMID: 23630581 PMC3632530

[ref42] Nübel U. Garcia-Pichel F. Muyzer G. (1997). PCR primers to amplify 16S rRNA genes from cyanobacteria. Appl. Environ. Microbiol. 63, 3327–3332. doi: 10.1128/aem.63.8.3327-3332.1997, PMID: 9251225 PMC168636

[ref43] Oksanen J. Blanchet F. G. Friendly M. Kindt R. Legendre P. McGlinn D.. (2020). Vegan: Community Ecology Package. R package version 2.5-7. Available at: https://CRAN.R-project.org/package=vegan

[ref44] Petersen R. (1991). Carbon-14 uptake by picoplankton and total phytoplankton in eight New Zealand lakes. Int. Rev. Gesamten Hydrobiol. Hydrogr. 76, 631–641. doi: 10.1002/iroh.19910760413

[ref45] Pick F. R. (2000). Predicting the abundance and production of photosynthetic picoplankton in temperate lakes. SIL Proc. 27, 1884–1889. doi: 10.1080/03680770.1998.11901568

[ref46] Pick F. (2016). Blooming algae: a Canadian perspective on the rise of toxic cyanobacteria. Can. J. Fish. Aquat. Sci. 73, 1149–1158. doi: 10.1139/cjfas-2015-0470

[ref47] Pulina S. Satta C. T. Padedda B. M. Bazzoni A. M. Sechi N. Lugliè A. (2017). Picophytoplankton seasonal dynamics and interactions with environmental variables in three Mediterranean coastal lagoons. Estuar. Coasts 40, 469–478. doi: 10.1007/s12237-016-0154-5

[ref48] Quast C. Pruesse E. Yilmaz P. Gerken J. Schweer T. Yarza P.. (2013). The SILVA ribosomal RNA gene database project: improved data processing and web-based tools. Nucleic Acids Res. 41, D590–D596. doi: 10.1093/nar/gks1219, PMID: 23193283 PMC3531112

[ref49] R Core Team (2021). R: A Language and Environment for Statistical Computing. Vienna, Austria: R Foundation for Statistical Computing. Available at: https://www.r-project.org/ (Accessed July 25, 2021).

[ref50] Rhew K. Baca R. M. Ochs C. A. Threlkeld S. T. (1999). Interaction effects of fish, nutrients, mixing and sediments on autotrophic picoplankton and algal composition: interaction effects on APP. Freshw. Biol. 42, 99–109. doi: 10.1046/j.1365-2427.1999.00464.x

[ref51] Ruber J. Bauer F. R. Millard A. D. Raeder U. Geist J. Zwirglmaier K. (2016). *Synechococcus* diversity along a trophic gradient in the Osterseen Lake District. Microbiology 162, 2053–2063. doi: 10.1099/mic.0.000389, PMID: 27902440

[ref52] Ruber J. Geist J. Hartmann M. Millard A. Raeder U. Zubkov M.. (2018). Spatio-temporal distribution pattern of the picocyanobacterium *Synechococcus* in lakes of different trophic states: a comparison of flow cytometry and sequencing approaches. Hydrobiologia 811, 77–92. doi: 10.1007/s10750-017-3368-z

[ref53] Sánchez-Baracaldo P. Bianchini G. Di Cesare A. Callieri C. Chrismas N. A. M. (2019). Insights into the evolution of picocyanobacteria and phycoerythrin genes (*mpeBA* and *cpeBA*). Front. Microbiol. 10:45. doi: 10.3389/fmicb.2019.00045, PMID: 30761097 PMC6363710

[ref54] Sanchez-Baracaldo P. Handley B. A. Hayes P. K. (2008). Picocyanobacterial community structure of freshwater lakes and the Baltic Sea revealed by phylogenetic analyses and clade-specific quantitative PCR. Microbiology 154, 3347–3357. doi: 10.1099/mic.0.2008/019836-0, PMID: 18957588

[ref55] Scanlan D. J. Ostrowski M. Mazard S. Dufresne A. Garczarek L. Hess W. R.. (2009). Ecological genomics of marine picocyanobacteria. Microbiol. Mol. Biol. Rev. 73, 249–299. doi: 10.1128/MMBR.00035-08, PMID: 19487728 PMC2698417

[ref56] Schallenberg M. (2020). The application of stressor–response relationships in the management of lake eutrophication. Inland Waters 11, 1–12. doi: 10.1080/20442041.2020.1765714

[ref57] Schallenberg M. Burns C. W. (2001). Tests of autotrophic picoplankton as early indicators of nutrient enrichment in an ultra-oligotrophic lake. Freshw. Biol. 46, 27–37. doi: 10.1046/j.1365-2427.2001.00647.x

[ref58] Schallenberg M. Larned S. T. Hayward S. Arbuckle C. (2010). Contrasting effects of managed opening regimes on water quality in two intermittently closed and open coastal lakes. Estuar. Coast. Shelf Sci. 86, 587–597. doi: 10.1016/j.ecss.2009.11.001

[ref59] Schallenberg L. A. Pearman J. K. Burns C. W. Wood S. A. (2021). Spatial abundance and distribution of picocyanobacterial communities in two contrasting lakes revealed using environmental DNA metabarcoding. FEMS Microbiol. Ecol. 97:fiab075. doi: 10.1093/femsec/fiab075, PMID: 34100943

[ref60] Schapira M. Buscot M.-J. Pollet T. Leterme S. C. Seuront L. (2010). Distribution of picophytoplankton communities from brackish to hypersaline waters in a south Australian coastal lagoon. Saline Syst. 6:2. doi: 10.1186/1746-1448-6-2, PMID: 20178652 PMC2847571

[ref61] Six C. Finkel Z. V. Irwin A. J. Campbell D. A. (2007). Light variability illuminates niche-partitioning among marine picocyanobacteria. PLoS One 2:e1341. doi: 10.1371/journal.pone.0001341, PMID: 18092006 PMC2129112

[ref62] Śliwińska-Wilczewska S. Maculewicz J. Barreiro Felpeto A. Latała A. (2018). Allelopathic and bloom-forming picocyanobacteria in a changing world. Toxins 10:48. doi: 10.3390/toxins10010048, PMID: 29361682 PMC5793135

[ref63] Śliwińska-Wilczewska S. Maculewicz J. Barreiro Felpeto A. Vasconcelos V. Latała A. (2017). Allelopathic activity of picocyanobacterium *Synechococcus* sp. on filamentous cyanobacteria. J. Exp. Mar. Biol. Ecol. 496, 16–21. doi: 10.1016/j.jembe.2017.07.008

[ref64] Sorokin Y. I. Dallocchio F. (2008). Dynamics of phosphorus in the Venice lagoon during a picocyanobacteria bloom. J. Plankton Res. 30, 1019–1026. doi: 10.1093/plankt/fbn059

[ref65] Stockner J. G. (1991). Autotrophic picoplankton in freshwater ecosystems: the view from the summit. Int. Rev. Gesamten Hydrobiol. Hydrogr. 76, 483–492. doi: 10.1002/iroh.19910760402

[ref66] Stockner J. G. Shortreed K. S. (1988). Response of *anabaena* and *Synechococcus* to manipulation of nitrogen: phosphorus ratios in a lake fertilization experiment: control of cyanobacteria blooms. Limnol. Oceanogr. 33, 1348–1361. doi: 10.4319/lo.1988.33.6.1348

[ref67] Stockner J. G. Shortreed K. S. (1991). Autotrophic picoplankton: community composition, abundance and distribution across a gradient of oligotrophic British Columbia and Yukon territory lakes. Int. Rev. Gesamten Hydrobiol. Hydrogr. 76, 581–601. doi: 10.1002/iroh.19910760410

[ref68] Szelag-Wasielewska E. (2004). Dynamics of autotrophic picoplankton communities in the epilimnion of a eutrophic Lake (Strzeszyńskie lake, Poland). Ann. Limnol. Int. J. Limnol. 40, 113–120. doi: 10.1051/limn/2004009

[ref69] Vila X. Abella C. A. (2001). Light-harvesting adaptations of planktonic phototrophic micro-organisms to different light quality conditions. Hydrobiologia 452, 15–30. doi: 10.1023/A:1011909330390

[ref70] Vörös L. Callieri C. Balogh K. V. Bertoni R. (1998). “Freshwater picocyanobacteria along a trophic gradient and light quality range.” in Phytoplankton Trophic Gradients. eds. Alvarez-Cobelas M. Reynolds C. S. Sánchez-Castillo P. Kristiansen J. (Dordrecht: Springer Netherlands), 117–125.

[ref71] Wang Q. Garrity G. M. Tiedje J. M. Cole J. R. (2007). Naïve Bayesian classifier for rapid assignment of rRNA sequences into the new bacterial taxonomy. Appl. Environ. Microbiol. 73, 5261–5267. doi: 10.1128/AEM.00062-07, PMID: 17586664 PMC1950982

[ref72] West N. J. Scanlan D. J. (1999). Niche-partitioning of *Prochlorococcus* populations in a stratified water column in the eastern North Atlantic Ocean. Appl. Environ. Microbiol. 65, 2585–2591. doi: 10.1128/AEM.65.6.2585-2591.1999, PMID: 10347047 PMC91382

[ref73] Wetzel R. G. Likens G. E. (1991). “Composition and biomass of phytoplankton.” in Limnol Anal. 2nd *Edn*. (New York, NY: Springer).

[ref74] Wickham H. (2016). Ggplot2: Elegant Graphics for Data Analysis. New York: Springer-Verlag.

[ref75] Winder M. (2009). Photosynthetic picoplankton dynamics in Lake Tahoe: temporal and spatial niche partitioning among prokaryotic and eukaryotic cells. J. Plankton Res. 31, 1307–1320. doi: 10.1093/plankt/fbp074

[ref76] Wood S. A. Maier M. Y. Puddick J. Pochon X. Zaiko A. Dietrich D. R.. (2017). Trophic state and geographic gradients influence planktonic cyanobacterial diversity and distribution in New Zealand lakes. FEMS Microbiol. Ecol. 93:fiw234. doi: 10.1093/femsec/fiw234 27856621

[ref77] Yang Y. Gu X. Te S. H. Goh S. G. Mani K. He Y.. (2019). Occurrence and distribution of viruses and picoplankton in tropical freshwater bodies determined by flow cytometry. Water Res. 149, 342–350. doi: 10.1016/j.watres.2018.11.022, PMID: 30469020

[ref78] Zhong X. Berdjeb L. Jacquet S. (2013). Temporal dynamics and structure of picocyanobacteria and cyanomyoviruses in two large and deep peri-alpine lakes. FEMS Microbiol. Ecol. 86, 312–326. doi: 10.1111/1574-6941.12166, PMID: 23772675

