# Supplementary material for: Metabarcoding Reveals Lacustrine Picocyanobacteria Respond to Environmental Change Through Adaptive Community Structuring
Source: Front Microbiol. 2021 Nov 12;12:757929. doi: 10.3389/fmicb.2021.757929 (PMC8633389; doi:10.3389/fmicb.2021.757929)
Supplement: Supplementary file 1 [file Image_1.pdf]

Supplementary Material

1 Supplementary information

Supplementary Table S1 – see separately attached .csv file.

Supplementary Table S2. Environmental variables removed from statistical analysis due to collinearity, bioenv selected variables for the CAP model and variance inflation factors for each selected variable used in this study. TN = total nitrogen, TP = total phosphorus, NNN = nitrate-nitrite nitrogen, DRP = dissolved reactive phosphorus, DO = dissolved oxygen, NH_4-N = ammoniacal nitrogen, Chl-*a* = Chlorophyll-*a*.

Lake	***Removed cross- correlated variables***	***Bioenv selected variables***	***Variance Inflation Factors***
Wanaka	Chl-*a*, NH_4-N, NNN and DRP	Temperature	1.9
		TN	2.5
		TP	2.1
		Secchi Depth	1.7
Wakatipu	DO, NH_4-N, TN, NNN and Chl-*a*	Secchi Depth	1.1
		TP	1.2
		Isothermal	1.0
Hayes	TP, TN, DRP, NH_4-N and DO	Secchi Depth	1.5
		Temperature	1.5
Tomahawk	Chl-*a*, DO, TP, Temperature and Salinity	TN	1.5
		NH_4-N	1.5
Ellesmere/ Te Waihora	Turbidity, TN and Secchi depth	DO	1.2
		Salinity	1.2

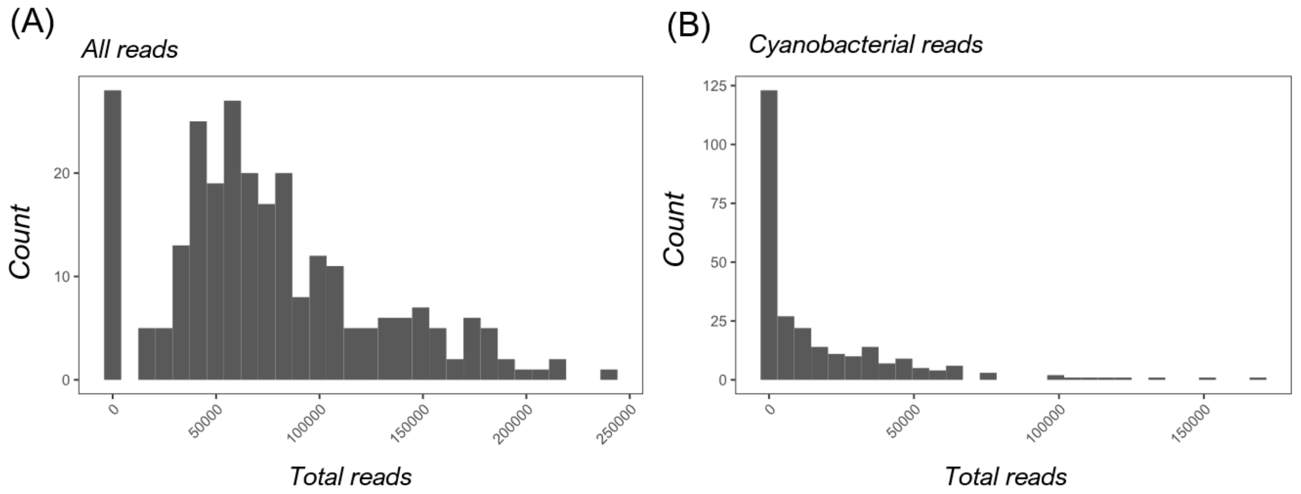

Supplementary Figure S1. Sequencing depth of (A) all reads retrieved from all samples, and (B) cyanobacterial reads from all samples.

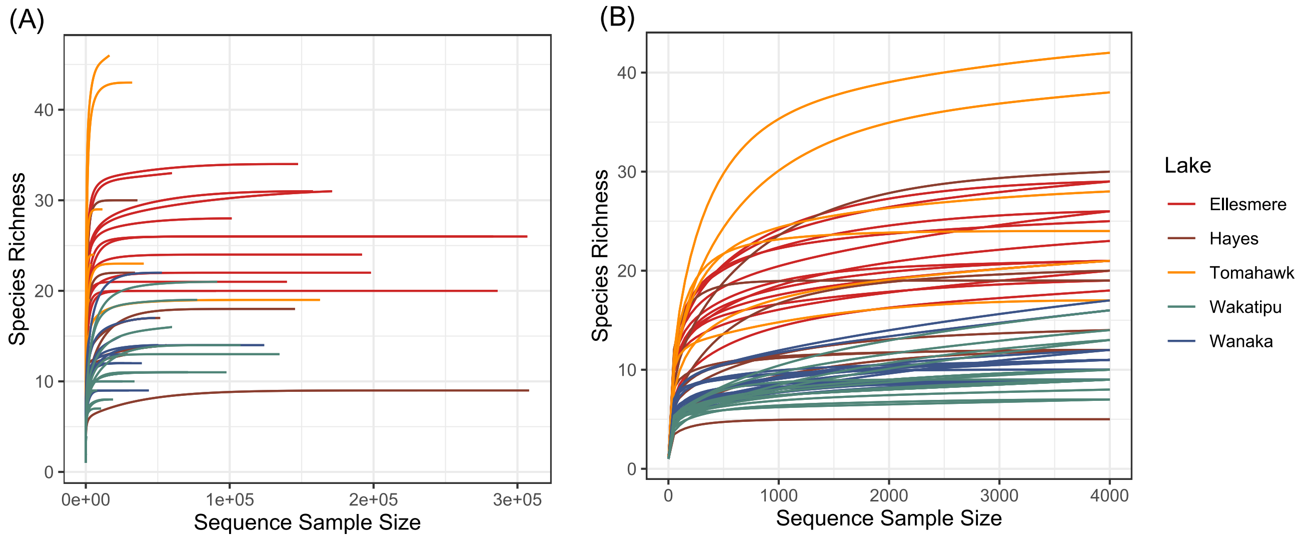

Supplementary Figure S2. Rarefaction curves of cyanobacterial reads in the five study lakes (A) before, and (B) after normalisation to 4000 reads.

Supplementary Table S3. Picocyanobacterial cell concentrations (cells/mL) across the five study lakes from February 2018 to February 2019. PE = phycoerythrin-rich, PC = phycocyanin-rich, Epi = epilimnion, Hyp = hypolimnion. Note all cells in Wanaka and Wakatipu were PE.

	Tomahawk				Wanaka				Wakatipu				Hayes								Ellesmere			
					Epi		Hyp		Epi		Hyp		Epi				Hyp							
	PE cells	PC cells	Total cell count	% colonial	Total cell count	% colonial	Total cell count	% colonial	Total cell count	% colonial	Total cell count	% colonial	PE cells	PC cells	Total cell count	% colonial	PE cells	PC cells	Total cell count	% colonial	Colonial	Single	Total cell count	% colonial
**Feb-18**	1715	45828	47543	96	14161	11	-	-	18010	18	-	-	45998	24223	70221	0	-	-	-	-	-	-	-	-
**Mar**	0	2412	2412	0	-	-	-	-	-	-	-	-	-	-	-	-	-	-	-	-	-	-	-	-
**Apr**	631	18612	19243	32	12876	12	-	-	19430	11	2412	0	-	-	-	-	-	-	-	-	5.68E+06	7.00E+06	1.27E+07	45
**May**	54	3270	3323	60	11524	29	1072	0	39878	16	2447	0	670	0	670	0	0	0	0	0	1.39E+07	1.15E+07	2.54E+07	55
**Jun**	59	5127	5186	44	17099	17	20421	56	35210	5	-	-	0	0	0	0	402	0	402	0	8.95E+06	8.25E+06	1.72E+07	52
**Jul**	408	3380	3788	34	16777	22	21011	28	34572	0	2734	0	0	0	0	0	0	0	0	0	1.28E+07	9.41E+06	2.22E+07	58
**Aug**	0	2913	2913	100	20603	37	15652	23	39342	9	35858	10	0	0	0	0	0	0	0	0	9.00E+06	3.68E+06	1.27E+07	71
**Sep**	117	1049	1166	0	40040	24	31181	24	33500	4	30659	15	0	0	0	0	0	0	0	0	1.46E+07	6.86E+06	2.14E+07	68
**Oct**	0	0	0	0	26854	29	10399	27	32374	4	23102	7	0	0	0	0	0	0	0	0	-	-	-	-
**Nov**	-	-	-	-	14794	19	6057	0	18921	2	20904	15	0	0	0	0	0	0	0	0	-	-	-	-
**Dec**	0	6176	6176	35	16402	21	7022	29	10077	13	9541	0	0	0	0	0	0	0	0	0	1.78E+07	8.33E+06	2.61E+07	68
**Jan**	0	2914	2914	100	20368	51	5682	36	16884	27	8469	0	20523	28693	49216	0	469	134	603	0	4.03E+06	6.06E+06	1.01E+07	40
**Feb-19**	-	-	-	-	20958	57	1769	0	12864	3	4020	0	30891	0	30891	0	603	0	603	0	1.19E+05	4.52E+07	4.52E+07	0

Supplementary Table S4. Number of amplicon sequence variants (ASVs) at each stage of data processing.

	Number of ASVs
Total recovered	13996
Cyanobacteria (class: Oxyphotobacteria)	5946
Cyanobacteria excluding chloroplasts	2515
Cyanobacteria post- filtering (>5 reads in >1 sample)	218
Cyanobacteria post- rarefaction (to 4000 reads)	206
Picocyanobacteria (order: Synechococcales)	68

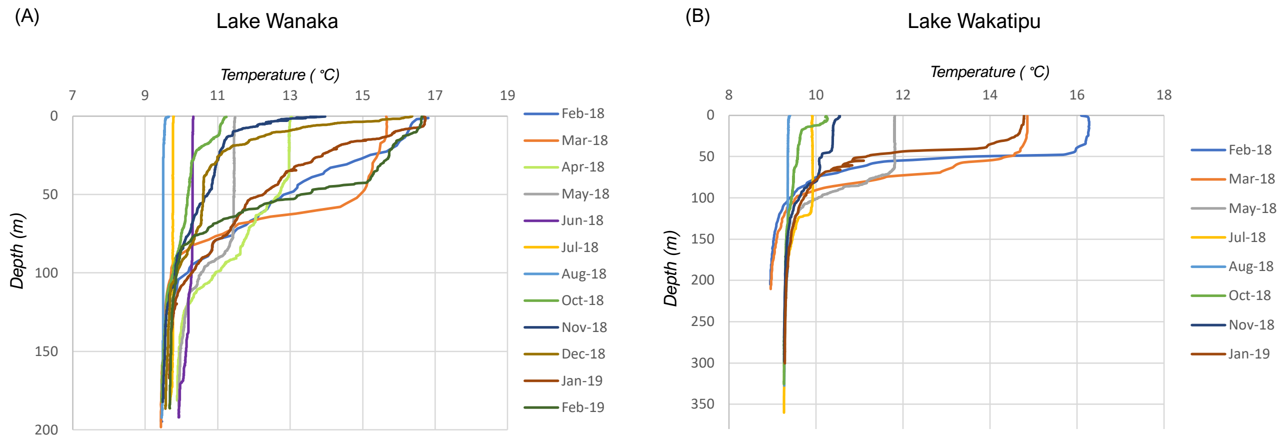

Supplementary Figure S3. Temperature depth profiles from (A) Lake Wanaka and (B) Lake Wakatipu measured over the study period (February 2018 to February 2019).

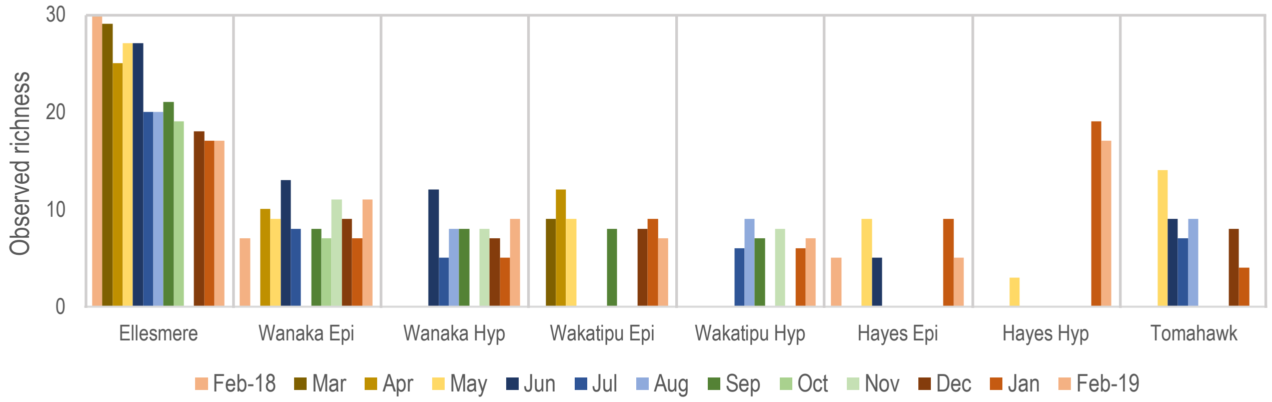

Supplementary Figure S4. Temporal observed richness of Picocyanobacteria Amplicon Sequence Variants in the five study lakes from February 2018 to February 2019 including hypolimnion and epilimnion samples from monomictic lakes. Blank bars indicate missing samples or samples removed during rarefaction.

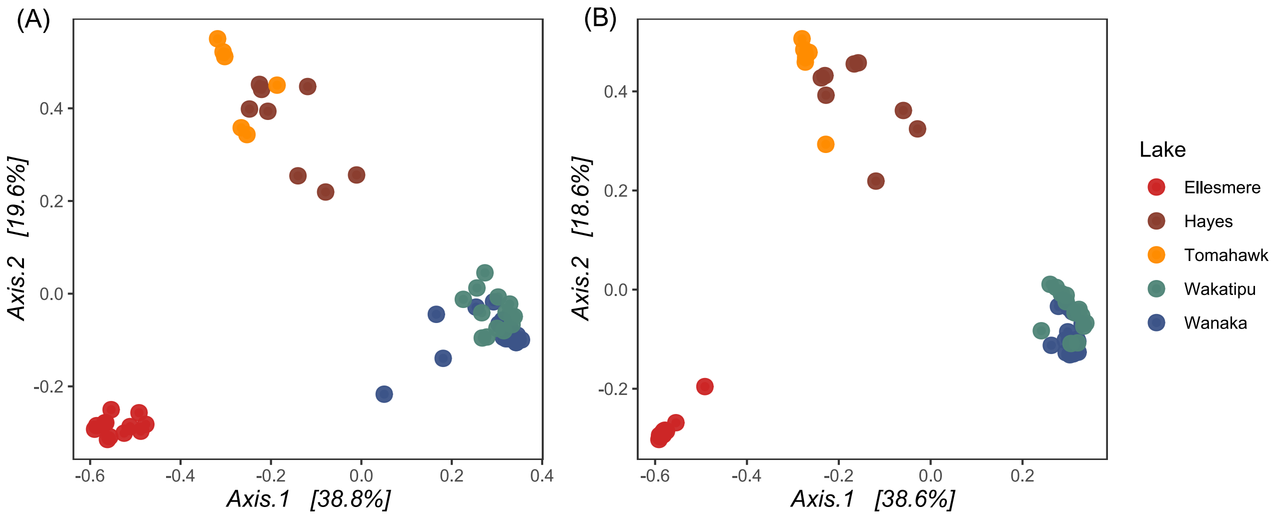

Supplementary Figure S5. Principal Co-ordinates Analysis of picocyanobacterial communities across the five study lakes using (A) binary Jaccard, and (B) weighted Jaccard dissimilarities.

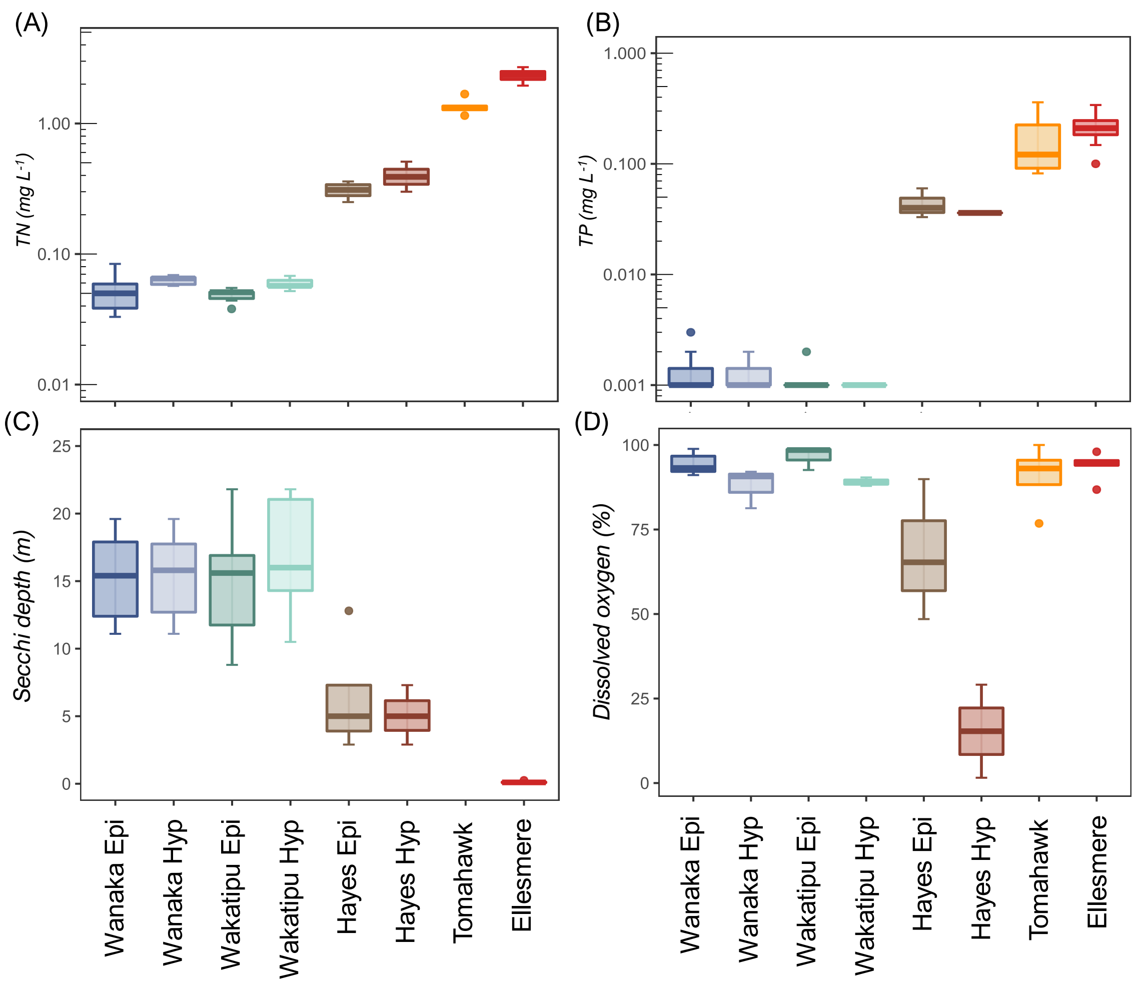

Supplementary Figure S6. Boxplots of environmental variables measured alongside samples collected from February 2018 to February 2019. Note the log scale in panels A and B. Epi = epilimnion, Hyp = hypolimnion. TN = total nitrogen, TP = total phosphorus.

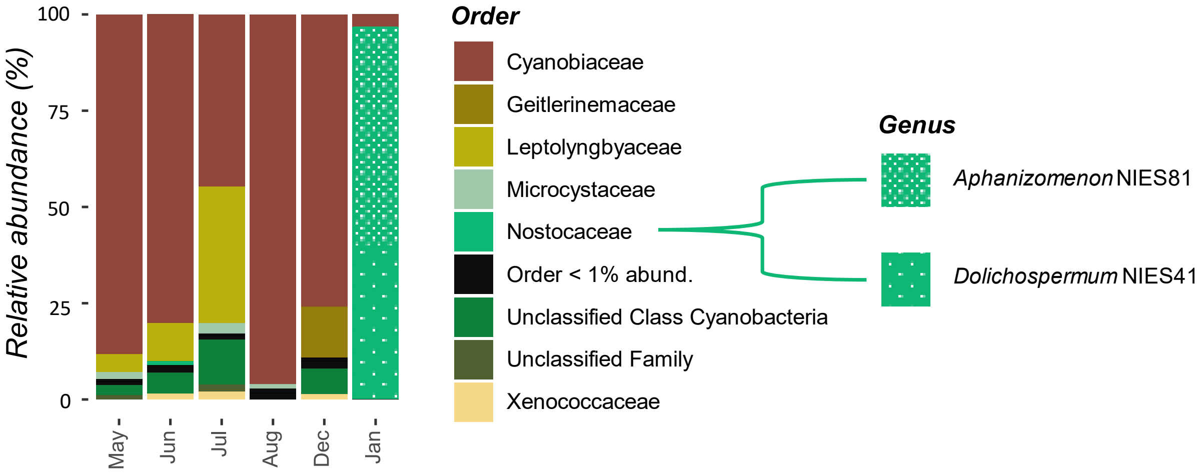

Supplementary Figure S7. Relative abundances of cyanobacterial orders found in Tomahawk Lagoon, with *Aphanizomenon* and *Dolichospermum* highlighted in January 2019.
